# Exome-wide analysis of rare coding variation identifies novel associations with COPD and airflow limitation in *MOCS3*, *IFIT3* and *SERPINA12*

**DOI:** 10.1136/thoraxjnl-2015-207876

**Published:** 2016-02-25

**Authors:** Victoria E Jackson, Ioanna Ntalla, Ian Sayers, Richard Morris, Peter Whincup, Juan-Pablo Casas, Antoinette Amuzu, Minkyoung Choi, Caroline Dale, Meena Kumari, Jorgen Engmann, Noor Kalsheker, Sally Chappell, Tamar Guetta-Baranes, Tricia M McKeever, Colin N A Palmer, Roger Tavendale, John W Holloway, Avan A Sayer, Elaine M Dennison, Cyrus Cooper, Mona Bafadhel, Bethan Barker, Chris Brightling, Charlotte E Bolton, Michelle E John, Stuart G Parker, Miriam F Moffat, Andrew J Wardlaw, Martin J Connolly, David J Porteous, Blair H Smith, Sandosh Padmanabhan, Lynne Hocking, Kathleen E Stirrups, Panos Deloukas, David P Strachan, Ian P Hall, Martin D Tobin, Louise V Wain

**Affiliations:** 1 Department of Health Sciences, University of Leicester, Leicester, UK; 2 William Harvey Research Institute, Barts and The London School of Medicine and Dentistry, Queen Mary University of London, London, UK; 3 Division of Respiratory Medicine, University of Nottingham, Queen's Medical Centre, Nottingham, UK; 4 School of Social & Community Medicine, University of Bristol, Bristol, UK; 5 Department of Primary Care & Population Health, UCL, London, UK; 6 Population Health Research Institute, St George's, University of London, London, UK; 7 University College London, Farr Institute of Health Informatics, London, UK; 8 Cochrane Heart Group, London, UK; 9 Department of Non-communicable Disease Epidemiology, Faculty of Epidemiology and Population Health, London School of Hygiene & Tropical Medicine, London, UK; 10 ISER, University of Essex, Colchester, Essex, UK; 11 Department of Epidemiology and Public Health, UCL, London, UK; 12 Institute of Cardiovascular Science, UCL, London, UK; 13 School of Life Sciences, University of Nottingham, Nottingham, UK; 14 Division of Epidemiology and Public Health, Nottingham City Hospital, University of Nottingham, Nottingham, UK; 15 Cardiovascular and Diabetes Medicine, School of Medicine, University of Dundee, Dundee, UK.; 16 Human Development & Health, Faculty of Medicine, University of Southampton, Southampton General Hospital, Southampton, UK; 17 NIHR Southampton Respiratory Biomedical Research Unit, University of Southampton and University Hospital Southampton NHS Foundation Trust, Southampton General Hospital, Southampton, UK; 18 MRC Lifecourse Epidemiology Unit, University of Southampton, Southampton General Hospital, Southampton, UK; 19 NIHR Southampton Biomedical Research Centre, University of Southampton and University Hospital Southampton NHS Foundation Trust, Southampton General Hospital, Southampton, UK; 20 Victoria University, Wellington, New Zealand; 21 Respiratory Medicine Unit, Nuffield Department of Medicine, University of Oxford, Oxford, UK; 22 Institute for Lung Health, Department of Infection, Immunity and Inflammation, University of Leicester, Leicester, UK; 23 National Institute for Health Research Respiratory Biomedical Research Unit, Glenfield Hospital, Leicester, UK; 24 Nottingham Respiratory Research Unit, University of Nottingham, City Hospital Campus, Nottingham, UK; 25 Institute for Ageing and Health, Newcastle University, Campus for Ageing and Vitality, Newcastle upon Tyne, UK; 26 Department of Molecular Genetics and Genomics, National Heart and Lung Institute, Imperial College London, London, UK; 27 Freemasons’ Department of Geriatric Medicine, University of Auckland, New Zealand; 28 Generation Scotland, Centre for Genomic and Experimental Medicine, Institute of Genetics and Molecular Medicine, University of Edinburgh, Edinburgh, UK; 29 Division of Population Health Sciences, University of Dundee, Dundee, UK; 30 Institute of Cardiovascular and Medical Sciences, University of Glasgow, Glasgow, UK; 31 Institute of Medical Sciences, University of Aberdeen, Aberdeen, UK; 32 Department of Haematology, University of Cambridge, Cambridge, UK; 33 Princess Al-Jawhara Al-Brahim Centre of Excellence in Research of Hereditary Disorders, King Abdulaziz University, Jeddah, Saudi Arabia

**Keywords:** COPD epidemiology, Tobacco and the lung

## Abstract

**Background:**

Several regions of the genome have shown to be associated with COPD in genome-wide association studies of common variants.

**Objective:**

To determine rare and potentially functional single nucleotide polymorphisms (SNPs) associated with the risk of COPD and severity of airflow limitation.

**Methods:**

3226 current or former smokers of European ancestry with lung function measures indicative of Global Initiative for Chronic Obstructive Lung Disease (GOLD) 2 COPD or worse were genotyped using an exome array. An analysis of risk of COPD was carried out using ever smoking controls (n=4784). Associations with %predicted FEV_1_ were tested in cases. We followed-up signals of interest (p<10^−5^) in independent samples from a subset of the UK Biobank population and also undertook a more powerful discovery study by meta-analysing the exome array data and UK Biobank data for variants represented on both arrays.

**Results:**

Among the associated variants were two in regions previously unreported for COPD; a low frequency non-synonymous SNP in *MOCS3* (rs7269297, p_discovery_=3.08×10^−6^, p_replication_=0.019) and a rare SNP in *IFIT3*, which emerged in the meta-analysis (rs140549288, p_meta_=8.56×10^−6^). In the meta-analysis of % predicted FEV_1_ in cases, the strongest association was shown for a splice variant in a previously unreported region, *SERPINA12* (rs140198372, p_meta_=5.72×10^−6^). We also confirmed previously reported associations with COPD risk at *MMP12, HHIP*, *GPR126* and *CHRNA5*. No associations in novel regions reached a stringent exome-wide significance threshold (p<3.7×10^−7^).

**Conclusions:**

This study identified several associations with the risk of COPD and severity of airflow limitation, including novel regions *MOCS3*, *IFIT3* and *SERPINA12*, which warrant further study.

**Key messages**

**What is the key question?**

- Do low frequency exonic variants influence susceptibility to COPD, and severity of airflow limitation?

**What is the bottom line?**

- Low frequency single nucleotide polymorphisms (SNPs) in *MOCS3 and IFIT3* were associated with risk of COPD and a rare splice variant in *SERPINA12* was associated with severity of airflow limitation.

**Why read on?**

- These genomic regions have not previously been implicated in lung function or COPD and these findings could therefore provide further insight into COPD susceptibility and severity.

## Introduction

COPD is a major public health concern, being a leading cause of morbidity and mortality worldwide.^1^ The Global Initiative for Chronic Obstructive Lung Disease (GOLD) recommends that the impact of COPD on an individual patient should assessed by considering breathlessness, symptoms and exacerbation risk, in combination with the severity of airflow limitation, which can be graded using %predicted FEV_1_.^2^ Approximately 1%–2% of COPD cases can be attributed to α1-antitrypsin (AAT) deficiency, a rare inherited disorder, caused by mutations within the *SERPINA1* gene.^3^ ^4^ For the remainder of COPD cases, cigarette smoking is recognised as the most significant risk factor^5^; however, there is also a genetic component, with several genomic regions showing association with COPD risk or airflow limitation to date, including *CHRNA3/5*, *HHIP,*^3^ *HTR4, GSTCD, TNS1,*^6^ *MMP12*^7^ ^8^ and *FAM13A.*^9^ COPD diagnosis is confirmed using measures of lung function, so it is likely that the genetic determinants of COPD and lung function will overlap. Indeed, many loci identified in large genome-wide association studies (GWAS) of FEV_1_ and the ratio of FEV_1_ to forced vital capacity (FEV_1_/FVC) in general population samples^10–13^ have subsequently being shown to be associated with COPD or airflow limitation.^6^ ^9^ ^14^ ^15^

Despite the successes in identifying genes associated with lung function and COPD, these known loci only explain a small proportion of the expected heritability.^13^ Large GWAS undertaken to date have generally focused on common variants (typically >5% minor allele frequency (MAF))^3^ ^9–14^; one hypothesis is that some of the so-called ‘missing heritability’ might be accounted for by variants of lower frequencies. In this study, we set out to investigate the role of low frequency, functional variants in COPD, and to confirm the role of single nucleotide polymorphisms (SNPs) previously showing association with lung function. It is hypothesised that rare variants are more likely than common variants to have deleterious effects; identifying such SNPs could lead to greater understanding of the pathways and biological mechanisms underlying airflow obstruction and COPD, and could translate to novel targets for treatment.

We genotyped cases with a history of smoking and airflow limitation, indicative of GOLD 2 COPD or worse, and control samples using an exome chip array to which we had added custom content comprising 2585 SNPs tagging regions which had shown suggestive association (p<2.21×10^−3^) with lung function in a previous large genome-wide HapMap-imputed study.^13^ The exome chip genotyping array design contains mostly non-synonymous, splice or stop codon altering variants that are likely to affect protein structure and function, with the majority of variants being low frequency (MAF 1%–5%) or rare (MAF<1%).

In this study, we carried out discovery case–control analyses (COPD cases vs controls) and analyses of %predicted FEV_1_ in cases, as a measure of severity of airflow limitation. Replication was undertaken using a subset of the UK Biobank Lung Exome Variant Evaluation (BiLEVE) study, a collection of 48 931 individuals from UK Biobank with high-quality lung function and smoking data who were genotyped on an array that includes substantial overlap with the exome chip.^16^ We also adopted a more powerful discovery strategy for COPD risk and severity of airflow limitation, by meta-analysing data for the subset of exome chip variants that were measured in both the COPD exome chip consortium and the UK BiLEVE study.

## Methods

### Study participants and phenotypes

A total of 3487 ever smokers with airflow limitation indicative of GOLD 2^2^ COPD or worse were identified from 12 UK collections as cases (case collections described in online supplementary table S1). Individuals met case criteria if they had FEV_1_/FVC ≤0.7 and %predicted FEV_1_ ≤80% (according to the National Health and Nutrition Examination Survey (NHANES) III spirometric reference equations^17^), did not have a doctor diagnosis of asthma and had reported current, or former smoking. Five of the sample collections (n=1398 samples, table 1) were COPD cohorts, with all individuals having irreversible airflow limitation, and meeting GOLD 2 criteria based on postbronchodilator spirometry. The remaining cases were taken from general population cohorts; for these samples, only prebronchodilator spirometry measures were available. We used general population controls with exome chip data, from Generation Scotland: Scottish Family Health Study (GS:SFHS), British 1958 Birth Cohort (1958BC), Oxford Biobank and GoDARTS (Genetics of Diabetes and Audit Research Tayside Study), listed in table 1 with clinical characteristics. All controls were current or former smokers and were free of lung disease, according to available spirometry and phenotype information.

10.1136/thoraxjnl-2015-207876.supp1 Supplementary data

**Table 1:** Clinical characteristics of samples passing genotype QC

		Sex	Age	%Predicted FEV_1_	FEV_1_/FVC	Pack-years	
Sample collection	n	Male, n (%)	Mean (SD)	Mean (SD)	Mean (SD)	Samples with data (n)	Mean (SD)
Discovery analyses airflow limitation cases (total n=3226, with pack-years n=2517)							
GS:SFHS	508	224 (44.1%)	58.9 (8.94)	64.84 (12.64)	0.580 (0.108)	482	29.32 (24.96)
British Regional Heart Study	425	425 (100%)	70.1 (5.46)	59.41 (14.66)	0.597 (0.084)	0	–
British Women's Heart and Health Study	254	0 (0%)	69.3 (5.46)	64.26 (12.40)	0.603 (0.074)	203	28.1 (18.36)
UK COPD cohort*	209	129 (61.7%)	68.7 (8.11)	37.94 (15.29)	0.447 (0.119)	199	50.07 (27.79
Hertfordshire Cohort Study	317	203 (64.0%)	66.1 (2.79)	62.89 (13.57)	0.589 (0.101)	312	32.25 (23.37)
COPDBEAT*	87	62 (71.3%)	67.6 (8.77)	45.19 (16.24)	0.480 (0.115)	86	38.69 (21.24)
Nottingham COPD study*	76	48 (63.2%)	67.2 (8.97)	50.29 (15.04)	0.482 (0.111)	74	49.02 (26.86)
Nottingham smokers	125	78 (62.4%)	63.1 (8.60)	46.27 (17.65)	0.503 (0.125)	124	41.75 (20.61)
Gedling study	33	26 (78.8%)	69.0 (8.23)	59.67 (16.81)	0.593 (0.103)	31	45.47 (33.40)
English Longitudinal Study of Aging	166	75 (45.2%)	66.0 (8.17)	54.84 (17.24)	0.526 (0.149)	0	–
EU COPD Gene Scan*	277	155 (56.0%)	67.0 (8.68)	38.51 (14.74)	0.467 (0.120)	277	46.43 (20.56)
GoTARDIS Study*	749	412 (55.0%)	68.8 (8.97)	52.16 (14.14)	0.509 (0.110)	729	43.26 (21.59)
Discovery analyses controls (total n=4784, with pack-years n=3889)							
GS:SFHS	961	552 (57.4%)	54.5 (8.41)	98.18 (10.92)	0.783 (0.051)	961	28.92 (16.86)
British 1958 Birth Cohort	1429	888 (62.1%)	44 (0)	100.90 (13.46)	0.809 (0.060)	1046	14.74 (10.07)
Oxford Biobank	1770	832 (47.0%)	41.6 (5.77)	–	–	1682	9.09 (9.34)
GoDARTS	624	402 (64.4%)	59.0 (10.75)	–	–	200	35.46 (25.89)
UK Biobank Lung Exome Variant Evaluation samples (meta-analysis and replication)							
Airflow limitation cases	4231	2379 (56.2%)	59.54 (6.86)	61.76 (11.8)	0.607 (0.076)	4231	42.41 (21.10)
Controls	8979	4260 (47.4%)	56.19 (7.92)	101.40 (8.1)	0.773 (0.038)	8979	30.43 (14.41)

*Sample collection is COPD case cohort.

GS:SFHS, Generation Scotland: Scottish Family Health Study; GoTARDIS, Tayside Allergy and Respiratory Disease Information System; QC, quality control.

We used a subset of the UK BiLEVE study^16^ for replication of novel signals, and for a larger discovery meta-analysis. A total of 24 457 heavy smokers (mean 35 pack-years) were genotyped as part of the UK BiLEVE study, selected such that 9748 individuals formed a low FEV_1_ group (based on %predicted FEV_1_), 4906 individuals formed a high FEV_1_ group and 9803 had average FEV_1_. We selected 4231 samples from the low FEV_1_ group, with airflow limitation consistent with GOLD 2 or worse as cases and 8979 samples from the high and average FEV_1_ groups with FEV_1_/FVC >0.7, %predicted FEV_1_ >80% and no doctor diagnosis of COPD for use as controls. All spirometry measures were prebronchodilator, all samples were heavy smokers and individuals with a doctor diagnosis of asthma or other lung diseases were excluded. The %predicted FEV_1_ was estimated using NHANES III spirometric reference equations.^17^

**Figure 1:**
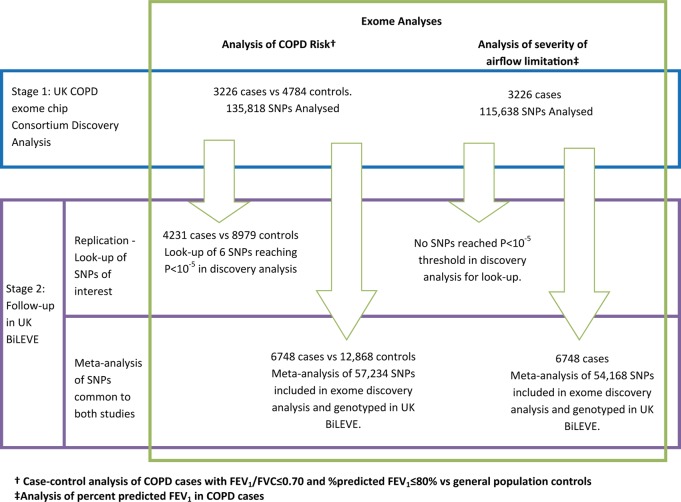
Two-stage study design. Stage 1: exome discovery analyses. Stage 2: Follow-up in UK BiLEVE: A. Replication of signals; B. meta-analysis of UK COPD exome chip consortium and UK BiLEVE.

An overview of the full study design is shown in figure 1.

### Genotyping

All 3487 cases and 1032 GS:SFHS controls were genotyped together using the Illumina Human Exome BeadChip with additional custom content for regions which have previously shown modest association with lung function (description of custom content design in online supplementary methods). The remaining discovery analyses control samples were genotyped separately using the Illumina Human Exome BeadChip.

The UK BiLEVE samples were genotyped using the Affymetrix UK BiLEVE array, which includes rare variants selected from the same sequencing project as the Illumina Human Exome BeadChip alongside additional content.^16^ Of the 807 411 SNPs included on the Affymetrix UK BiLEVE array, 74 891 were also present on the Illumina Human Exome BeadChip; this subset of SNPs, which were directly genotyped on both arrays, was selected for the discovery meta-analysis.

### Quality control of genotype data

#### Discovery exome analysis

Genotypes were called using Illumina's Gencall algorithm in Genomestudio^18^ with refinement of rare variants with missing calls undertaken using zCall.^19^ Standard quality control (QC) filters were applied, in accordance with the Exome-chip Quality Control SOP V.5, as developed within the UK exome chip consortium^20^ and are fully described in online supplementary methods. In brief, SNPs were excluded if they had low call rate (<99%) or deviated from Hardy Weinberg Equilibrium (p<10^−4^) and samples were excluded if they were duplicates, sex mismatches, heterozygosity outliers (>3 SD from mean), had an excess of singleton SNPs, or were ancestral outliers. Clusterplots for all SNPs of interest were inspected, to ensure accuracy of genotype calling.

#### UK BiLEVE data

The QC procedure of the UK BiLEVE genotype data is described elsewhere.^16^

### Statistical analyses

SNP associations with COPD risk were carried out using a logistic regression model, adjusting for age, sex and pack-years and assuming an additive genetic model. Associations with untransformed %predicted FEV_1_ in cases were tested, using a linear regression model, with adjustment for pack-years (analysis of severity of airflow limitation). Since not all samples had pack-years data available, secondary analyses were carried out without adjustment for pack-years, for both the COPD risk and severity of airflow limitation analyses, allowing the inclusion of all samples. Single variant analyses were carried out using PLINK V.1.07.^21^ Using a Bonferroni correction for the number of tests undertaken, a significance level of p<3.7×10^−7^ would be required in the exome single variant analysis to retain a type 1 error of 5%. We defined SNPs of interest as those with p<10^−5^ in the discovery exome analysis; for these SNPs, we undertook replication analyses in the UK BiLEVE study to corroborate findings (see online supplementary methods). We set a Bonferroni corrected significance level for replication, for the number of SNPs in novel loci taken forward to replication (p<0.017 for analysis of COPD risk). Gene-based analyses using SKAT-O were additionally undertaken; the methods and results of these analyses are described in the online supplementary information.

### Custom content single variant analyses

Custom content comprising 2585 SNPs tagging regions which had shown suggestive association (p<2.21×10^−3^) with lung function in a previous large genome-wide HapMap-imputed study^13^ were also included on the array for cases and GS:SFHS controls. Additional controls from 1958BC and Busselton Health Study (BHS) with genome-wide data were also used; full methods and results of this analysis are given in the supplementary information.

### Meta-analysis with UK BiLEVE data

Single variant associations with COPD risk and severity of airflow limitation in the UK BiLEVE samples were carried out using PLINK v1.07,^21^ identically to the corresponding discovery analysis with pack-years adjustment. We carried out an inverse-variance–weighted meta-analysis of the union of SNPs included in the discovery exome and UK BiLEVE analyses (described in online supplementary methods).

## Results

### Discovery exome analysis

3226 cases and 4784 controls passed all sample and SNP genotype QC and were used in the exome analysis (exclusions in online supplementary table S1). Clinical characteristics of these samples are summarised in table 1. Of the SNPs which passed all QC criteria in both cases and controls, 135 818 were polymorphic, of which 101 308 (74.6%) had a MAF<1%.

#### Analyses of COPD risk

**Figure 2:**
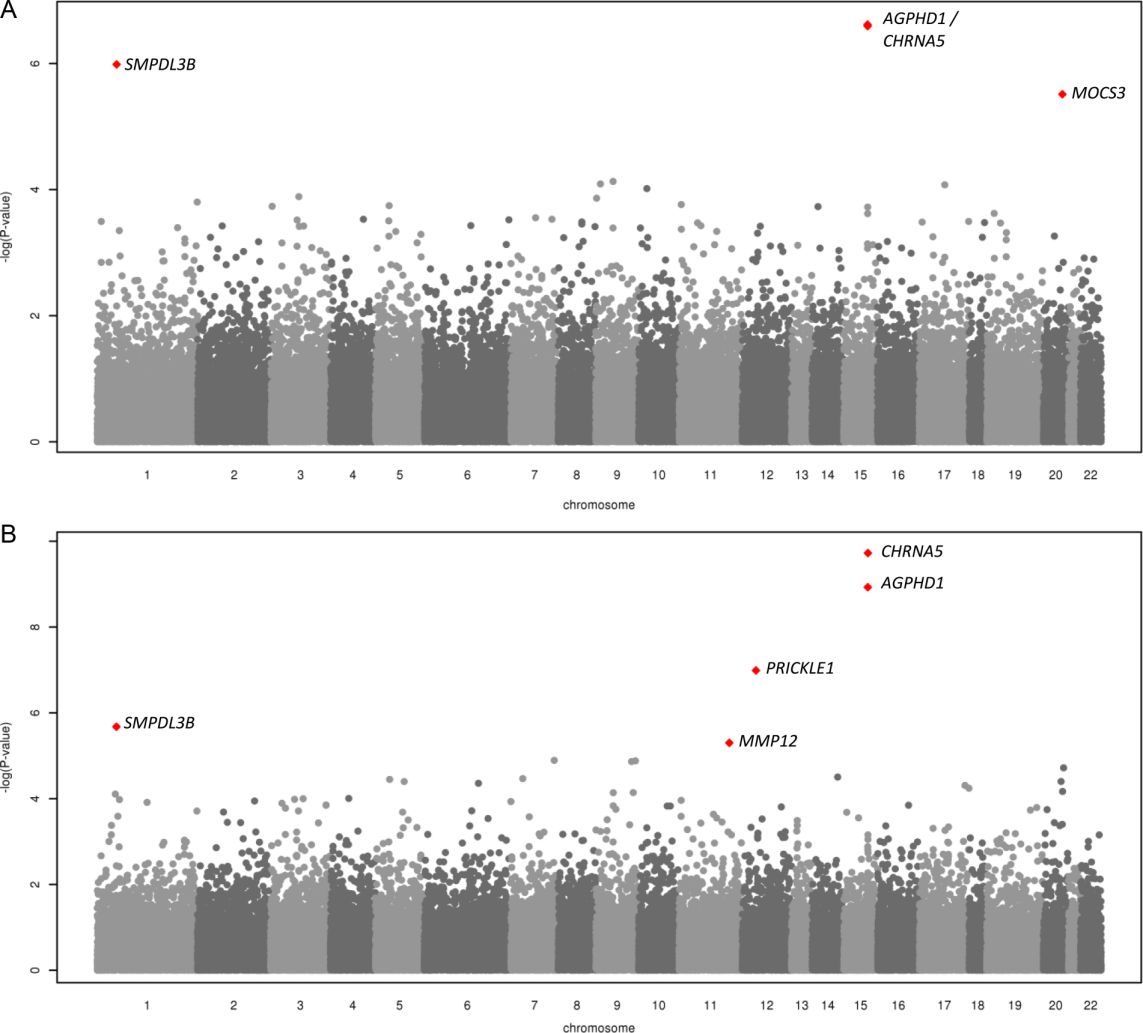
(A) Analysis of COPD risk, with pack-years adjustment (single nucleotide polymorphisms (SNPs) with minor allele frequency (MAF) >0.05% only; SNPs with p<10^−5^ highlighted). (B) Analysis of COPD risk, without pack-years adjustment (SNPs with MAF >0.05% only; SNPs with p<10^−5^ highlighted).

We carried out pack-years adjusted analysis of COPD risk, including 2517 cases and 3889 controls, in addition to an unadjusted analysis, using all 3226 cases and 4784 controls (quantile–quantile plots shown in online supplementary figure S1). A total of four SNPs in three regions met the p<10^−5^ significance threshold in the pack-years adjusted analysis, with five SNPs in four regions showing p<10^−5^ in the unadjusted analysis (figure 2).

In the pack-years adjusted analysis (table 2A and figure 2A), the most significant association was for the previously reported COPD/smoking region 15q25 (sentinel SNP rs8034191 OR: 1.38, MAF=34.8%, p=2.42×10^−7^). This signal was replicated in the UK BiLEVE study. Two novel signals of association with COPD risk (p<10^−5^) were rs3813803 within *SMPDL3B* (OR: 1.37, MAF=29.2%, p=1.04×10^−6^) and low frequency SNP rs7269297 within *MOCS3* (OR: 0.25, MAF=1.1%, p=3.08×10^−6^). There was evidence of replication, just above the Bonferroni corrected level of significance (p<0.017) for rs7269297 in the UK BiLEVE study (p=7.27×10^−5^ for meta-analysis of discovery and UK BiLEVE results, table 2A).

**Table 2:** Top associations in exome discovery analyses and meta-analysis of COPD risk

(A) SNPs with p<10^–5^ in either the pack-years adjusted or unadjusted discovery analyses																		
					Discovery pack-years adjusted analysis (2517 cases, 3889 controls)				Discovery unadjusted analysis (3226 cases, 4784 controls)				UK BiLEVE pack-years adjusted analysis (4231 cases, 8979 controls)				Meta-analysis of discovery and UK BiLEVE pack-year adjusted analyses	
					MAF (MAC)		Association result		MAF (MAC)		Association result		MAF (MAC)		Association result		Association result	
rs no.	CHR	Position	Coded allele	Gene	Cases	Controls	OR (95% CI)	p Value*	Cases	Controls	OR (95% CI)	p Value*	Cases	Controls	OR (95% CI)	p Value*	OR (95% CI)	p Value*
rs3813803	1	28282292	C	*SMPDL3B* (non-synonymous)	30.6% (1541)	28.3% (2203)	1.370 (1.207 to 1.554)	**2.41×10^−6^**	30.3% (1956)	28.5% (2722)	1.288 (1.160 to 1.430)	**2.11×10^−6^**	28.7% (2418)	29.4% (5269)	0.968 (0.911 to 1.029)	0.298	1.033 (0.978 to 1.092)	0.241
rs17368582	11	102738075	C	*MMP12* (synonymous)	11.1% (561)	12.9% (1001)	0.767 (0.642 to 0.915)	3.22×10^−3^	11.1% (719)	12.8% (1229)	0.712 (0.615 to 0.824)	**5.01×10^−6^**	12.0% (1015)	12.2% (2198)	0.982 (0.902 to 1.069)	0.676	0.938 (0.868 to 1.013)	0.101
rs3827522	12	42853871	A	*PRICKLE1* (non-synonymous)	0.2% (11)	0.4% (27)	0.184 (0.065 to 0.519)	1.39×10^−3^	0.2% (14)	0.5% (46)	0.123 (0.057 to 0.266)	**1.03×10^−7^**	0.3% (21)	0.3% (45)	0.907 (0.518 to 1.585)	0.731	0.633 (0.386 to 1.039)	0.071
rs8034191	15	78806023	C	near *AGPHD1* (intergenic)	38.0% (1912)	32.7% (2546)	1.374 (1.218 to 1.550)	**2.42×10^−7^**	37.7% (2432)	32.9% (3144)	1.364 (1.234 to 1.507)	**1.18×10^−9^**	39.2% (3315)	35.2% (6320)	1.156 (1.092 to 1.224)	**6.85×10^−7^**	1.193 (1.133 to 1.257)	**2.79×10^−11^**
rs7269297	20	49576664	G	*MOCS3* (non-synonymous)	0.7% (37)	1.4% (110)	0.251 (0.140 to 0.448)	**3.08×10^−6^**	0.8% (54)	1.5% (139)	0.423 (0.262 to 0.680)	3.98×10^−4^	1.2% (98)	1.4% (252)	0.742 (0.578 to 0.953)	0.019	0.626 (0.497 to 0.789)	7.27×10^−5^

(B) SNPs with p<10^–5^ in the meta-analysis (only most statically significant SNP in each region shown)														
					Discovery pack-years adjusted analysis (2517 cases, 3889 controls)				UK BiLEVE pack-years adjusted analysis (4231 cases, 8979 controls)				Meta-analysis of discovery and UK BiLEVE pack-year adjusted analyses	
					MAF (MAC)		Association result		MAF (MAC)		Association result		Association result	
rs no.	CHR	Position	Coded allele	Gene	Cases	Controls	OR (95% CI)	p Value*	Cases	Controls	OR (95% CI)	p Value*	OR (95% CI)	p Value*
rs1828591	4	145480780	A	GYPA/HHIP (intergenic)	35.6% (1794)	39.1% (3042)	0.9167 (0.814, 1.032)	0.153	36.6% (3088)	40.0% (771)	0.867 (0.819, 0.918)	**9.88×10^−7^**	0.876 (0.832, 0.922)	**5.75×10^−7^**
rs4896582	6	142703877	A	GPR126 (intronic)	29.3% (1473)	31.7% (2468)	0.8594 (0.757, 0.974)	0.018	28.0% (2349)	30.2% (5344)	0.879 (0.826, 0.934)	3.87×10^−5^	0.875 (0.827, 0.925)	**2.53×10^−6^**
rs140549288	10	91099466	C	IFIT3 (exonic), LIPA (intronic)	0.8% (38)	0.6% (44)	2.156 (1.046, 4.445)	0.037	0.9% (79)	0.6% (100)	1.880 (1.378, 2.565)	6.87×10^−5^	1.924 (1.441, 2.560)	**8.56×10^−6^**

*p Values in bold significant at p<**10^−5^** level.

BiLEVE, Biobank Lung Exome Variant Evaluation; MAC, minor allele count; MAF, minor allele frequency; SNPs, single nucleotide polymorphisms.

A further two loci were associated with COPD risk in the analysis unadjusted for pack-years: rs3827522 within *PRICKLE1* (OR: 0.12, MAF=0.4%, p=1.03×10^−7^) and rs17368582 within *MMP12* (OR: 0.712, MAF=12.2% p=5.01×10^−6^, table 2A and figure 2B); however, there was no evidence of replication of these associations with COPD risk in UK BiLEVE. rs2276109, another SNP within *MMP12*, (MAF=5.6%) which is strongly correlated with rs17368582 (r^2^=0.84), has previously been associated with COPD risk in smokers.^7^ Overall, no associations in novel regions met exome-wide significance (p<3.7×10^−7^).

#### Analyses of severity of airflow limitation

Although no SNPs reached the p<10^−5^ significance level in either the pack-years adjusted, or the unadjusted analysis (see online supplementary figures S2 and S3), six SNPs showed some evidence of association (p<10^−4^) in one or both analyses (see online supplementary table S2). Of note, rs28929474, the z-allele within the *SERPINA1* gene, showed modest association in the unadjusted analysis (β=−6.17%, MAF=2.0%, p=2.83×10^−5^).

### UK BiLEVE meta-analysis results

#### Analyses of COPD risk

**Figure 3:**
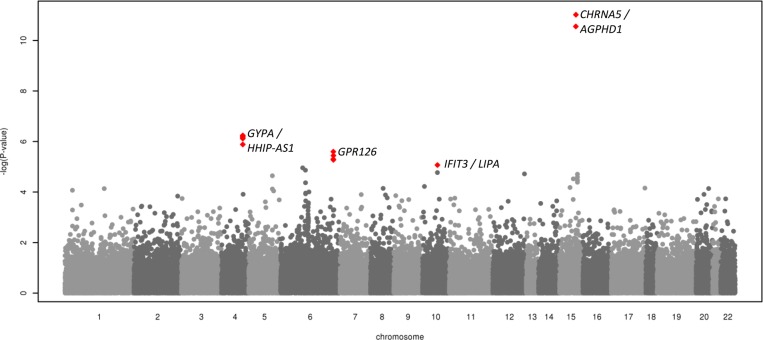
Meta-analysis of COPD risk in discovery exome analysis and UK Biobank Lung Exome Variant Evaluation samples.

For the 57 234 polymorphic SNPs common to both the COPD exome chip consortium samples and the UK BiLEVE study, a meta-analysis of discovery and UK BiLEVE study results was undertaken in which three regions showed association with risk of COPD (p<10^−5^, figure 3, online supplementary figure S4 and table 2B). The *GYPA/HHIP* and *GPR126* regions have previously been reported as showing association with lung function and COPD or airflow limitation risk.^3^ ^10^ ^14^ The *IFIT3* region signal (rs140549288 p.Val352Leu in *IFIT3*, OR: 1.92, MAF=0.7%, p=7.49×10^−6^) represents a novel rare variant signal of association with COPD.

#### Analyses of severity of airflow limitation

A total of 54 168 SNPs were included in the meta-analysis of severity of airflow limitation (see online supplementary figures S5 and S6). One SNP showed association with p<10^−5^: rs140198372, a variant which alters the sequence at a site where the splicing of an intron takes place (splice site) in *SERPINA12* (β=−33.51%, MAF=0.03%, p=5.72×10^−6^, table 3).

**Table 3:** Top associations (p<10^−5^) in meta-analysis of severity of airflow limitation

					Severity of airflow limitation, adjusted for pack-years (n=2517)			UK BiLEVE pack-years adjusted analysis (n=4231)			Meta-analysis of discovery and UK BiLEVE pack-year adjusted analyses	
rs no.	CHR	Position	Coded allele	Gene	MAF (MAC)	Beta (95% CI)	p Value	MAF (MAC)	Beta (95% CI)	p Value	Beta (95% CI)	p Value
rs140198372	14	94953832	A	*SERPINA12* (splice site)	0.059% (3)	−29.23 (−49.50 to −8.96)	2.59×10^−5^	0.012% (1)	−38.35 (−59.88 to −16.82)	4.11×10^−4^	−33.51 (−48.27 to −18.76)	**5.72×10^−6^**

*p Values in bold significant at p<10^−5^ level.

BiLEVE, Biobank Lung Exome Variant Evaluation; MAC, minor allele count; MAF, minor allele frequency.

### Sensitivity analyses to assess COPD case criteria

Of our 3226 COPD cases defined as described above, 1398 also had a GOLD 2 or worse COPD based on postbronchodilator spirometry. We carried out a sensitivity analysis for all SNPs identified in our discovery or meta-analyses of COPD risk, by repeating the discovery analyses including only those 1398 COPD cases which underwent reversibility testing. This analysis showed consistent estimated effect sizes (see online supplementary table S3 and figure S7), and in particular, the ORs were not substantially attenuated for rs7269297 in *MOCS3* (sensitivity analysis OR: 0.276; original discovery OR: 0.251), nor rs140549288 in *IFIT3* (sensitivity analysis OR: 2.554; original discovery OR: 2.156).

### Association of novel loci with smoking behaviour

Given the disparity of smoking behaviour in our cases and control samples (table 1), we further investigated whether either of the two novel COPD risk loci were associated with smoking behaviour, to ascertain whether the associations with COPD may be explained by differences in smoking. Neither of the sentinel SNPs showed significant association with heavy versus never smoking within UK BiLEVE (p=0.956 for rs7269297 and p=0.945 for rs140549288) study. We further undertook a look-up in the publically available results of a GWAS from the Tobacco and Genetics consortium^22^ for associations with rs7269297 in *MOCS3* (rs140549288 was not available in data) and a number of smoking traits; however, no evidence for association with smoking behaviour was found (cigarettes per day p=0.610; ever vs never smoking p=0.172; current vs former smoking p=0.699).

## Discussion

We carried out analyses of exome chip variants with COPD risk and %predicted FEV_1_ among cases, through which we identified a number of SNPs in both known COPD regions and at novel loci that showed suggestive association (p<10^−5^) with risk of COPD. These novel regions (region plots: online supplementary figure S8) warrant further investigation as they may provide insight into the underlying biological mechanisms of COPD and airflow limitation in smokers and could provide novel therapeutic targets. The most significant associations in both the discovery exome analysis and the meta-analysis were with SNPs in the 15q25 region, previously identified through GWAS as being associated with smoking behaviour,^22–24^ lung cancer,^25^ COPD^3^ and airflow obstruction.^14^ In addition, we independently replicated previously reported associations of *HHIP,*^3^ ^10^ *GPR126*^14^ and *MMP12*^7^ ^8^ with COPD risk.

We identified novel associations between COPD risk and low frequency or rare coding SNPs in two genes: *MOCS3* (rs7269297, serine to alanine, MAF=1.3%, p_discovery_=3.08×10^−6^, PolyPhen prediction: benign) and *IFIT3* (rs140549288, valine to leucine, MAF=0.7%, p_meta_=8.56×10^−6^, PolyPhen prediction: benign). The protein encoded by *MOCS3* adenylates and activates molybdopterin synthase, an enzyme required to synthesise molybdenum cofactor^26^ and is expressed in bronchial epithelium and smooth muscle layer of the bronchus.^27^ *IFIT3* is associated with interferon-α antiviral activity and has been found to be up-regulated in respiratory syncytial virus infection^28^ and in human lung epithelial cells infected with dengue virus.^29^ The SNP rs140549288 is also located within in an intron of *LIPA*; the product of this gene is involved in the hydrolysis of cholesteryl esters and triglycerides and other SNPs within this gene have previously been associated with coronary artery disease.^30^

The z-allele within the *SERPINA1* gene was associated with a lower %predicted FEV_1_ in cases (unadjusted analysis: p_discovery_=2.83×10^−5^); as well as being a well-established cause of AAT deficiency,^3^ ^4^ this SNP has also previously been associated with an increased annual decline in FEV_1_ in a general population sample^31^ and increased airflow limitation in COPD cases.^32^ In the present study, the z-allele was associated with an increased risk of COPD, although this was not statistically significant (OR: 1.27, p=0.252). The likely reason for the lack of a significant association with this known COPD locus is that some of the case collections excluded individuals with AAT deficiency, resulting in selection bias. In the meta-analysis of severity of airflow limitation, we identified a very rare SNP within another serine protease inhibitor gene, *SERPINA12*, not previously associated with COPD (rs140198372, MAF=0.03%, p_meta_=5.72×10^−6^). SERPINA12 and SERPINA1 lie 96.6 kb apart on chromosome 14 (rs140198372 and the z-allele in SERPINA1 are not in linkage disequilibrium (r2=9.0×10^−6^)). *SERPINA12* has been associated with cardiovascular diseases, being implicated in obesity and type 2 diabetes.^33^

One of the primary challenges associated with identifying low frequency variants associated with disease is limited statistical power, and this could explain our lack of strong statistically significant findings. Indeed, none of the reported associations in novel regions met a stringent exome-wide significance level (p<3.8×10^−7^) overall. In the present study, we would have just 54% power to detect an association with an SNP associated with COPD risk with a MAF of 1% and an OR of 2, at the p<3.8×10^−7^ level. Furthermore, recent analyses undertaken by the UK10K Consortium found no evidence of low frequency SNPs having large effects, upon a series of traits.^34^ Due to the limited power to detect single variant associations of rare variants with modest effect sizes, we additionally adopted gene-based analyses using SKAT-O, a method which combines information from several rare variants (see online supplementary information). In these analyses, we only identified one gene meeting our elected significance level (p<10^−5^); this gene-based signal in *PRICKLE1* was found however, to be driven by a single SNP, which was identified as being associated with COPD risk in the single variant discovery analysis, but which was not replicated in the UK BiLEVE data.

Another limitation of this study is that a number of our cases had only prebronchodilator spirometry; for these samples, it could not be determined whether their airflow limitation was reversible, and so a proportion of these cases may not have met the clinical definition of COPD. We undertook case–control sensitivity analyses using our discovery samples, restricting cases to the subset of 1398 individuals taken from COPD cohorts and who had known irreversible airflow limitation. The effect estimates of our top hits did not substantially change in this sensitivity analysis, suggesting that our broader case definition, including samples that did not undergo reversibility testing, did not result in substantial misclassification bias.

A further potential source of bias in this study was the heavier smoking history in our cases compared with the control samples. For the two SNPs identified through the analyses of COPD risk, we found no evidence of association with smoking in data from the UK BiLEVE study, suggesting that the associations with COPD risk were not driven by the imbalances in smoking behaviour.

Finally, it was not possible to validate the findings of this study through additional genotyping; however for the three reported loci, consistent results were observed in both the discovery and the UK BiLEVE samples. It would not be expected to see the same false positive result in these two independent samples, therefore, strengthening the evidence for these being true associations.

In summary, we have identified potentially interesting associations with low frequency and rare SNPs and COPD risk in two regions not previously implicated in COPD or lung function. We further identified an association of %predicted FEV_1_ in individuals with COPD with a very rare SNP in *SERPINA12*. Further confirmation of these associations in larger independent collections of COPD cases and controls is needed. This study also provides further evidence that the z-allele within *SERPINA1* may be related to severity of airflow limitation in COPD. While large sample sizes may be required to definitively identify novel loci, we present evidence to support the notion that the genetic contribution to COPD risk comprises polygenic contributions of rare, low frequency and common genetic variants. Future studies, alone or in combination, should aim to target the full allele frequency range to unravel the genetic architecture of COPD.
