# Supplementary material for: Exome-wide analysis of rare coding variation identifies novel associations with COPD and airflow limitation in MOCS3, IFIT3 and SERPINA12
Source: Thorax. 2016 Feb 25;71(6):501–9. doi: 10.1136/thoraxjnl-2015-207876 (PMC4893124; doi:10.1136/thoraxjnl-2015-207876)
Supplement: Supplementary data [file thoraxjnl-2015-207876supp.pdf]

Supplementary Information

Exome-wide analysis of rare coding variation identifies novel associations with COPD and airflow limitation in *MOCS3*, *IFIT3* and *SERPINA12*.

Victoria E Jackson[1], Ioanna Ntalla[1,2], Ian Sayers[3], Richard Morris[4,5], Peter Whincup[6], Juan-Pablo Casas[7,8], Antoinette Amuzu[9], Minkyoung Choi[9], Caroline Dale[9], Meena Kumari[10,11], Jorgen Engmann[12], Noor Kalsheker[13], Sally Chappell[13], Tamar Guetta-Baranes[13], Tricia M McKeever[14], Colin NA Palmer[15], Roger Tavendale[15], John W Holloway[16,17], Avan A Sayer[18,19], Elaine M. Dennison[18,20], Cyrus Cooper[18,19], Mona Bafadhel[21], Bethan Barker[22,23], Chris Brightling[22,23], Charlotte E Bolton[24], Michelle E John[24], Stuart G Parker[25], Miriam F Moffat[26], Andrew J Wardlaw[22,23], Martin J Connolly[27], David J Porteous[28], Blair H Smith[29], Sandosh Padmanabhan[30], Lynne Hocking[31], Kathleen E Stirrups[2,32], Panos Deloukas[2,33],David P. Strachan[6], Ian P. Hall[3], Martin D Tobin[1,23] Louise V Wain[1]

1. Department of Health Sciences, University of Leicester, Leicester LE1 7RH, UK
2. William Harvey Research Institute, Barts and The London School of Medicine and Dentistry, Queen Mary University of London, London, UK
3. Division of Respiratory Medicine, University of Nottingham, Queen’s Medical Centre, Nottingham NG7 2UH, UK
4. School of Social & Community Medicine, University of Bristol, Bristol, UK, BS8 2PS
5. Dept of Primary Care & Population Health, UCL, London, UK, NW3 2PF
6. Population Health Research Institute, St George's, University of London, Cranmer Terrace, London SW17 0RE, UK
7. University College London, Farr Institute of Health Informatics, London, UK
8. Cochrane Heart Group, London, UK
9. Department of Non-communicable Disease Epidemiology, Faculty of Epidemiology and Population Health, London School of Hygiene & Tropical Medicine, London, WC1E 7HT, United Kingdom
10. ISER, University of Essex, Colchester, Essex. UK. CO4 3SQ
11. Department of Epidemiology and Public Health, UCL, London. UK. WC1E 6BT
12. Institute of Cardiovascular Science, UCL, London. UK. WC1E 6BT
13. School of Life Sciences, University of Nottingham, UK
14. Division of Epidemiology and Public Health, Nottingham City Hospital, University of Nottingham, Nottingham,NG5 1PB
15. Cardiovascular and Diabetes Medicine, School of Medicine, University of Dundee, Dundee, DD1 9SY, UK.
16. Human Development & Health, Faculty of Medicine, University of Southampton, Southampton General Hospital, Southampton SO16 6YD, UK
17. NIHR Southampton Respiratory Biomedical Research Unit, University of Southampton and University Hospital Southampton NHS Foundation Trust, Southampton General Hospital, Southampton SO16 6YD, UK
18. MRC Lifecourse Epidemiology Unit, University of Southampton, Southampton General Hospital, Southampton SO16 6YD, UK
19. NIHR Southampton Biomedical Research Centre ,University of Southampton and University Hospital Southampton NHS Foundation Trust, Southampton General Hospital, Southampton SO16 6YD, UK
20. Victoria University, Wellington, New Zealand.
21. Respiratory Medicine Unit, Nuffield Department of Medicine, University of Oxford, Oxford, UK OX3 7FZ
22. Institute for Lung Health, Department of Infection, Immunity, and Inflammation, University of Leicester, Leicester, UK
23. National Institute for Health Research Respiratory Biomedical Research Unit, Glenfield Hosptial, Leicester
24. Nottingham Respiratory Research Unit, University of Nottingham, City Hospital Campus, Hucknall road, Nottingham. NG5 1PB
25. Institute for Ageing and Health, Newcastle University, Campus for Ageing and Vitality, Newcastle upon Tyne NE4 5PL, UK
26. Department of Molecular Genetics and Genomics, National Heart and Lung Institute, Imperial College London, London, United Kingdom

27. Freemasons' Department of Geriatric Medicine, University of Auckland, New Zealand
28. Generation Scotland, Centre for Genomic and Experimental Medicine, Institute of Genetics and Molecular Medicine, University of Edinburgh, Edinburgh, UK EH4 2XU
29. Division of Population Health Sciences, University of Dundee, Dundee UK DD2 4R
30. Institute of Cardiovascular and Medical Sciences, University of Glasgow, Glasgow UK G12 8TA
31. Institute of Medical Sciences, University of Aberdeen, Aberdeen, UK, AB25 2ZD
32. Department of Haematology, University of Cambridge, Cambridge, UK
33. Princess Al-Jawhara Al-Brahim Centre of Excellence in Research of Hereditary Disorders, King Abdulaziz University, Jeddah, Saudi Arabia;

Contents

Supplementary Tables 3
Supplementary Figures 6
Supplementary methods 12
  Case Collections 13
  Genotype calling and Quality Control (QC) of exome array genotype data 15
  Discovery exome gene-based analysis..... 16
  Custom content design, genotyping and QC of genotype data 16
  Custom content single variant analyses 18
  Replication and meta-analysis with UK BiLEVE data..... 18
Supplementary Results 19
  Discovery exome gene-based analysis..... 19
  Custom content single variant association analysis 21

Supplementary Tables

Supplementary Table 1: Genotype QC for samples used in exome analyses.

	Cases	GS:SFHS Controls	1958BC Controls*	OXBB Controls*	GoDARTS Controls*
**Initial sample**	**3487**	**1032**	**-**	**-**	**-**
**Samples failing stage 1 QC: pre zCall**					
Call rate < 90%	34	1	-	-	-
Sex mismatches	18	2	-	-	-
Heterozygosity outliers (common SNPs MAF ≥ 1%)	41	3	-	-	-
Heterozygosity outliers (rare SNPs MAF < 1%)	28	6	-	-	-
Call rate < 98%	43	46	-	-	-
Duplicates (PI_HAT > 0.95)	56	0	-	-	-
PCA outliers (+/- 3SD of the mean)	12	2	-	-	-
Samples with excess number of singletons SNPs (> 50)	15	6	-	-	-
Inconsistency with GWAS data	1	0	-	-	-
XY-intensity outliers (+/- 4SD of the mean)	12	0	-	-	-
**Samples passing pre zCall QC**	**3302**	**976**	**1456**	**1822**	**635**
**Samples failing stage 2 QC: post zCall**					
Call rate < 99%	0	0	0	0	0
Heterozygosity outliers	76	15	27	52	11
**Final Samples Passing both QC stages**	**3226**	**961**	**1429**	**1770**	**624**

***Stage 1 QC and recalling of genotypes using zCall carried out for 1958BC, OXBB and GoDARTs controls within UK exome chip consortium.**

Supplementary Table 2: Top associations in exome analysis and meta-analysis of severity of airflow limitation.

a. **SNPs with $P<10^{-4}$ in either the pack-years adjusted, or unadjusted discovery analyses**

					Severity of airflow limitation, adjusted for pack-years (n=2517)			Unadjusted analysis of severity of airflow limitation (n=3226)			UK BiLEVE pack-years adjusted analysis (n=4231)			Meta-analysis of discovery and UK BiLEVE pack-year adjusted analyses.	
rs no.	CHR	Position	Coded Allele	Gene	MAF (MAC)	Beta	P	MAF (MAC)	Beta	P	MAF (MAC)	Beta	P	Beta	P
rs77108843	19	1828148	A	*REXO1* (nonsynonymous)	0.50% (25)	13.37	7.44×10^{-5}	0.59% (38)	12.09	1.18×10^{-5}	0.82% (63)	-0.741	0.590	0.057	0.396
rs59035258	8	65527669	T	*CYP7B1* (nonsynonymous)	3.7% (187)	4.178	8.75×10^{-4}	3.6% (232)	4.793	2.11×10^{-5}	4.1% (314)	-0.224	0.721	0.386	0.306
rs117991621	12	96379884	T	*HAL* (nonsynonymous)	0.56% (28)	10.92	6.19×10^{-4}	0.56%[3] (36)	12.02	2.26×10^{-5}	0.50% (42)	-1.221	0.569	0.133	0.423
rs28929474	14	94844947	T	*SERPINA1* (nonsynonymous)	2.2% (109)	-5.053	1.30×10^{-3}	2.0% (127)	-6.165	2.83×10^{-5}	2.2% (183)	-1.53	0.061	-2.583	2.73×10^{-3}
rs11749	1	38023316	T	*DNALI1* (nonsynonymous)	24.1% (1212)	-1.57	3.99×10^{-3}	24.3% (1565)	-1.967	4.30×10^{-5}	25.1% (2123)	-0.3228	0.235	-1.789	0.027
rs147487857	15	41247629	G	*CHAC1* (nonsynonymous)	1.3% (63)	-8.895	3.27×10^{-5}	1.3% (81)	-5.619	3.19×10^{-3}	1.5% (123)	0.2003	0.840	-0.597	0.163

Supplementary Table 3: Sensitivity analysis of SNPs identified in either the discovery, or meta-analyses of COPD risk. Results for original analyses, and for analyses where cases restricted to include only those with known irreversible airflow limitation

a. SNPs identified in discovery analyses

					Discovery pack-years adjusted analysis (2517 cases, 3889 controls)		Discovery pack-years adjusted analysis (1365 COPD cases with reversibility testing, 3889 controls)		Discovery unadjusted analysis (3226 cases, 4784 controls)		Discovery unadjusted analysis (1398 COPD cases with reversibility testing, 4784 controls)	
rs no.	CHR	Position	Coded Allele	Gene	OR	P*	OR	P*	OR	P*	OR	P*
rs3813803	1	28282292	C	*SMPDL3B* (nonsynonymous)	1.37	**2.41x10^{-6}**	1.46	**5.18 x10^{-6}**	1.288	**2.11 x10^{-6}**	1.382	**2.98 x10^{-6}**
rs17368582	11	102738075	C	*MMP12* (synonymous)	0.767	3.22 x10^{-3}	0.673	1.08 x10^{-3}	0.712	**5.01 x10^{-6}**	0.6567	2.35 x10^{-5}
rs3827522	12	42853871	A	*PRICKLE1* (nonsynonymous)	0.184	1.39 x10^{-3}	0.272	1.08 x10^{-3}	0.123	**1.03 x10^{-7}**	0.1836	1.43 x10^{-4}
rs8034191	15	78806023	C	near *AGPHD1* (intergenic)	1.374	**2.42 x10^{-7}**	1.42	**8.14 x10^{-6}**	1.364	**1.18 x10^{-9}**	1.414	**1.33 x10^{-7}**
rs7269297	20	49576664	G	*MOCS3* (nonsynonymous)	0.251	**3.08 x10^{-6}**	0.276	4.05 x10^{-4}	0.423	3.98 x10^{-4}	0.4502	0.0118

b. SNPs identified in meta-analyses

					Discovery pack-years adjusted analysis (2517 cases, 3889 controls)		Discovery pack-years adjusted analysis (1365 COPD cases with reversibility testing, 3889 controls)		Discovery unadjusted analysis (3226 cases, 4784 controls)		Discovery unadjusted analysis (1398 COPD cases with reversibility testing, 4784 controls)	
rs no.	CHR	Position	Coded Allele	Gene	OR	P*	OR	P*	OR	P*	OR	P*
rs1828591	4	145480780	A	GYPA / HHIP (intergenic)	0.9167	0.153	0.8727	0.08661	0.919	0.093	0.8821	0.05665
rs4896582	6	142703877	A	GPR126	0.8594	0.018	0.8676	0.08605	0.864	5.95 x10^{-3}	0.8702	0.04272
rs140549288	10	91099466	C	IFIT3 (exonic), LIPA (intronic)	2.156	0.037	2.554	0.06401	1.823	0.057	2.211	0.04798

***P-values in bold significant at P<10^{-5} level**

Supplementary Figures

Supplementary Figure 1: Quantile-quantile plots for analyses of COPD risk with (right) and without (left) pack-years adjustment. SNPs with MAF>0.05% only.

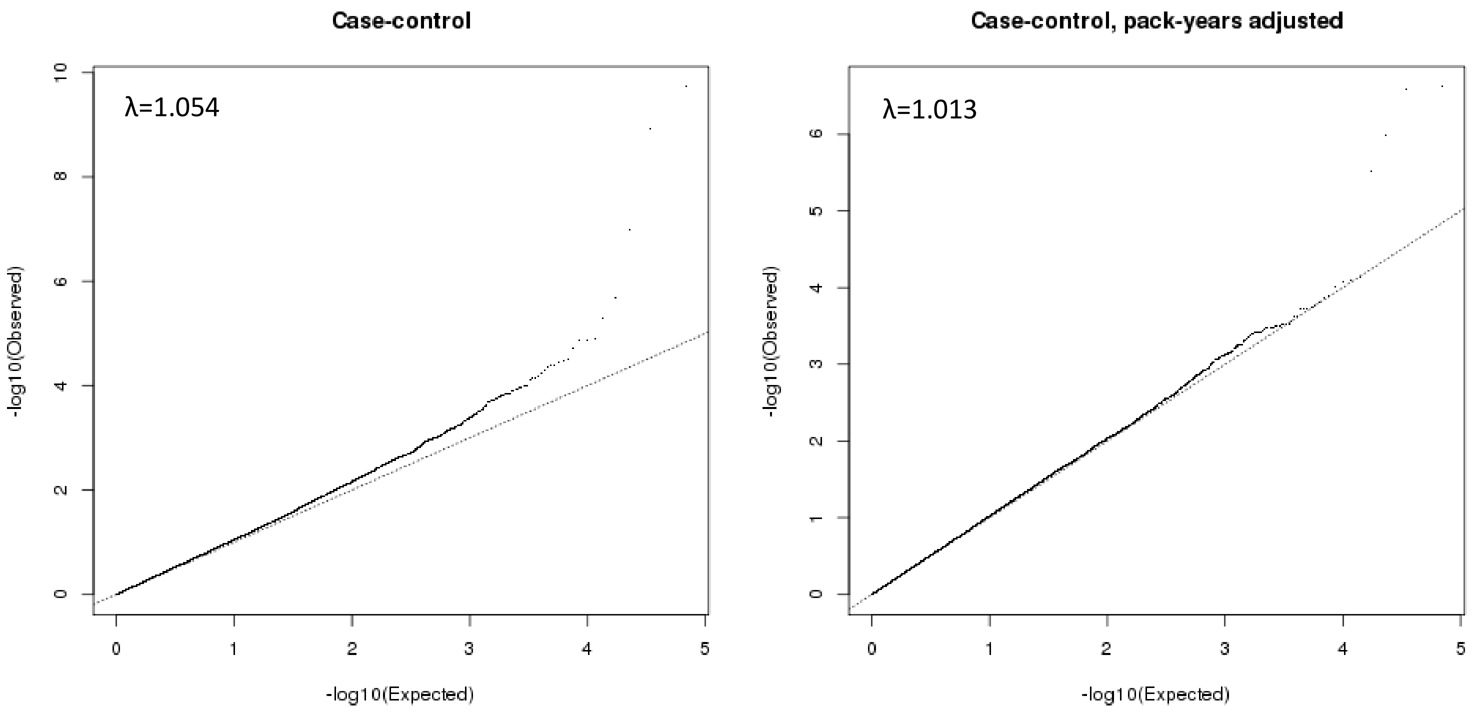

Supplementary Figure 2: Quantile-quantile plots for analyses of severity of airflow limitation with (left) and without (right) pack-years adjustment. Plots include all SNPs passing genotype QC.

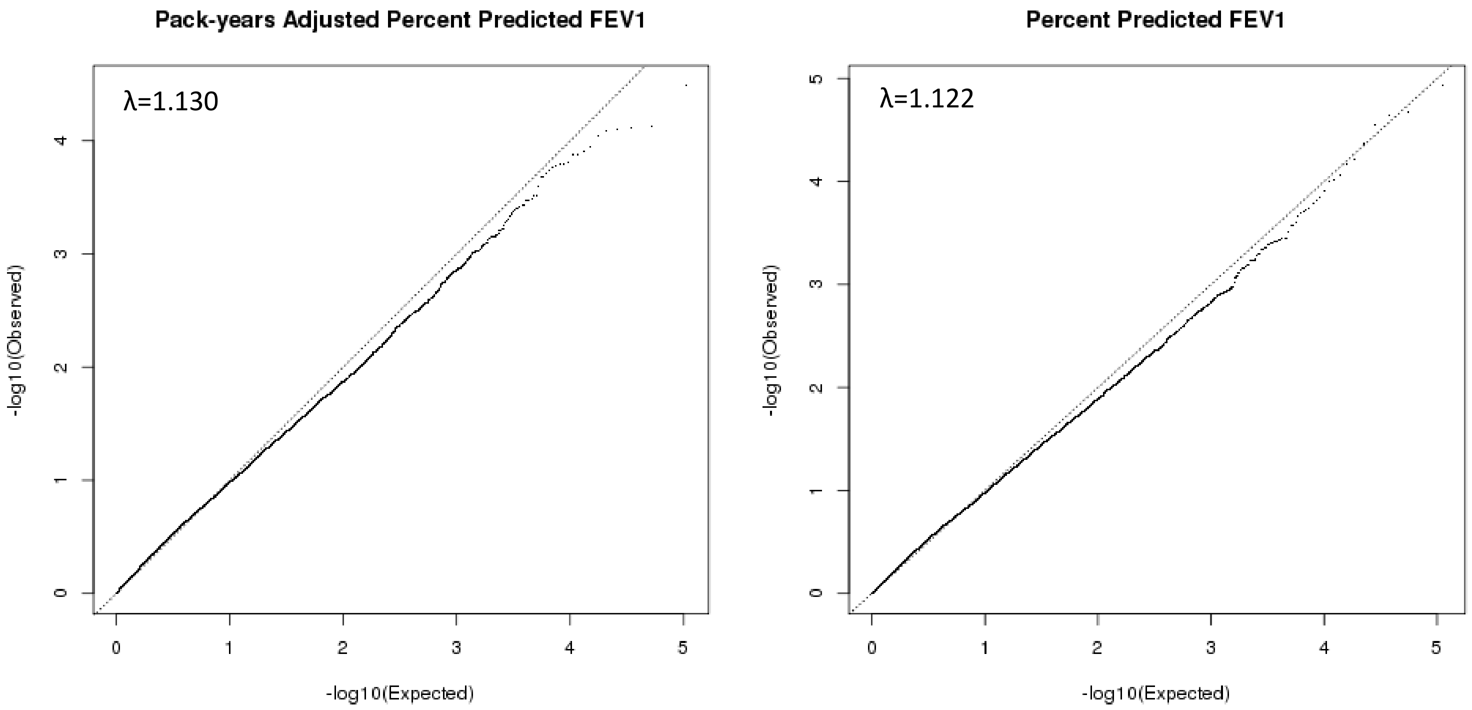

Supplementary Figure 3: A) Manhattan for severity of airflow limitation analysis, adjusted for pack-years smoking (all SNPs passing genotype QC). B)Manhattan for severity of airflow limitation analysis without adjustments for pack-years smoking (all SNPs passing genotype QC).

A)

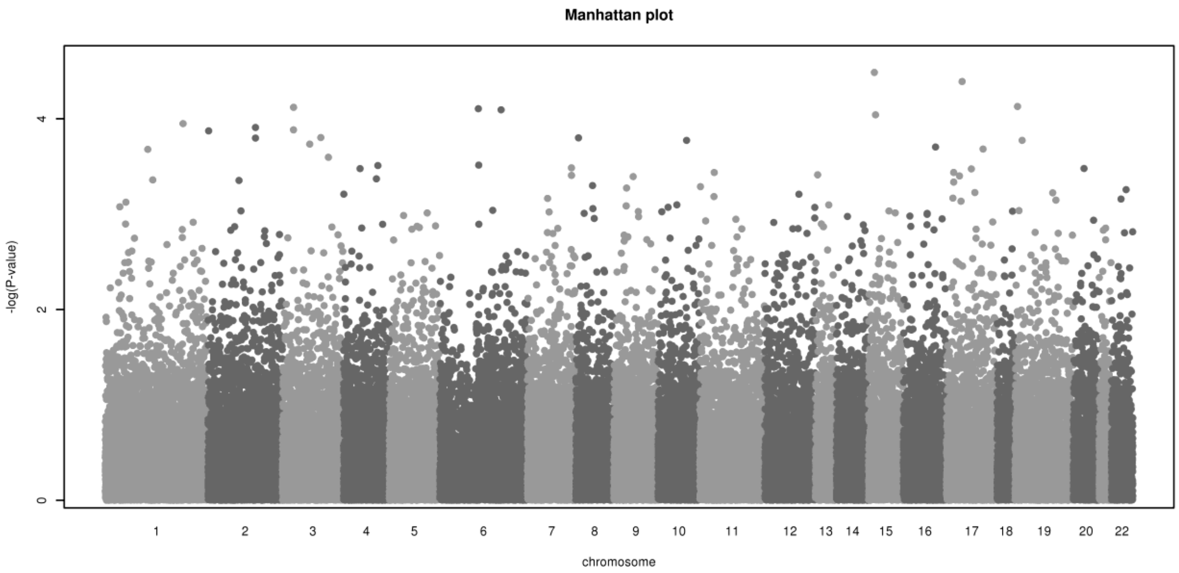

B)

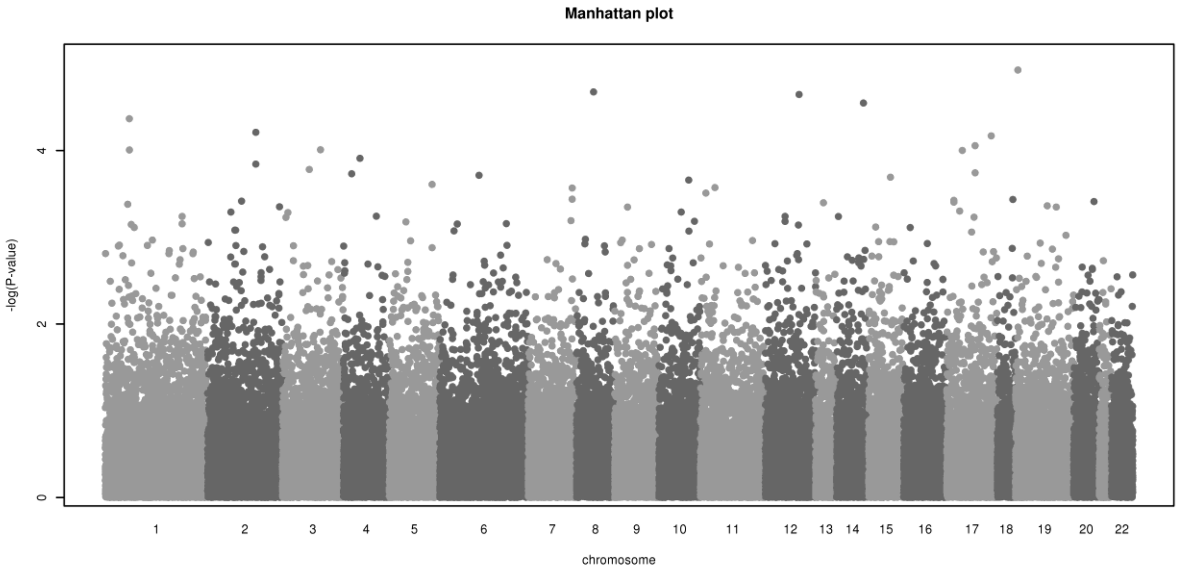

Supplementary Figure 4: Quantile-quantile plots for meta-analysis of COPD risk in discovery exome analysis and UK BiLEVE samples.

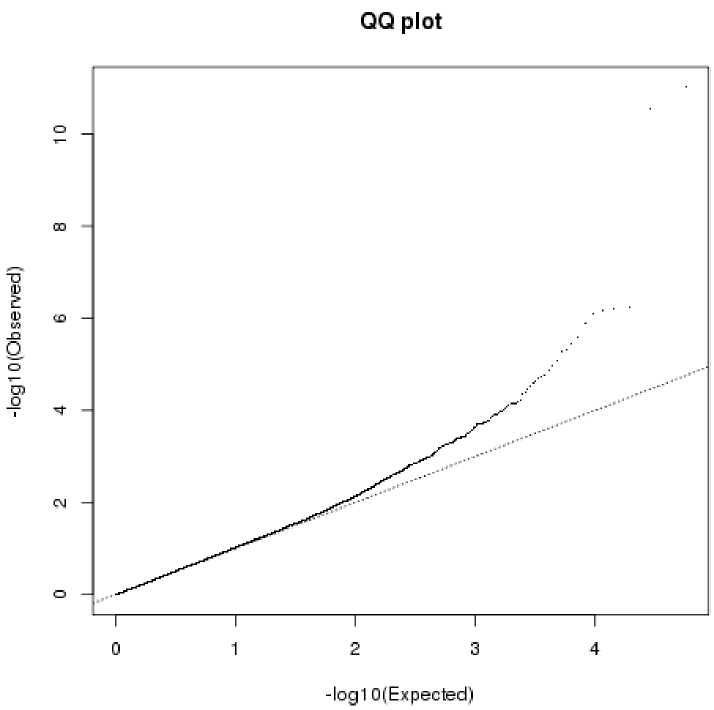

Supplementary Figure 5: Quantile-quantile plots for meta-analysis of severity of airflow limitation in discovery exome analysis and UK BiLEVE samples

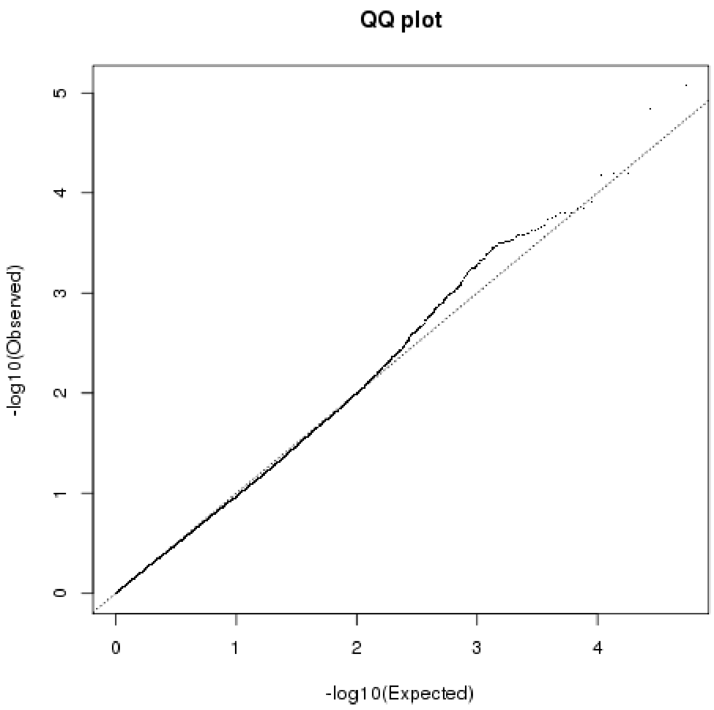

Supplementary Figure 6: Manhattan plot for meta-analysis of severity of airflow limitation in discovery exome analysis and UK BiLEVE samples

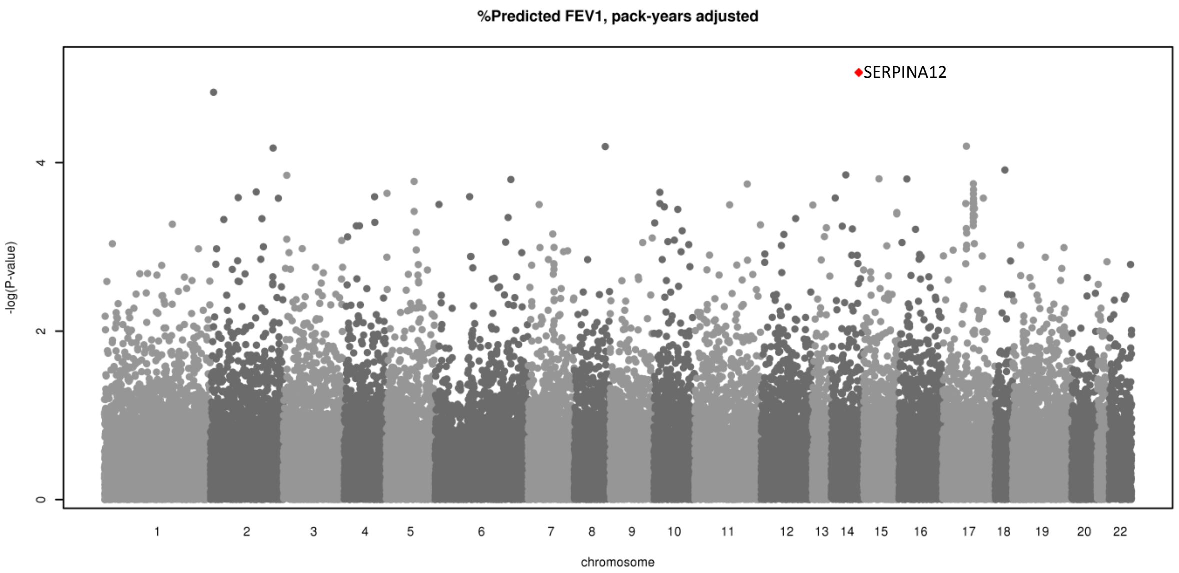

Supplementary Figure 7: Comparison of effect estimates of discovery case-control analysis of COPD risk where the cases were restricted to only include those with known irreversible airflow obstruction, versus the analysis including all COPD cases. Highlighted are the effect estimates of the two SNPs we report in novel regions (rs7269297 in *MOCS3* and rs140549288 in *IFIT3*).

B. Discovery analysis of COPD risk, adjusted for pack-years

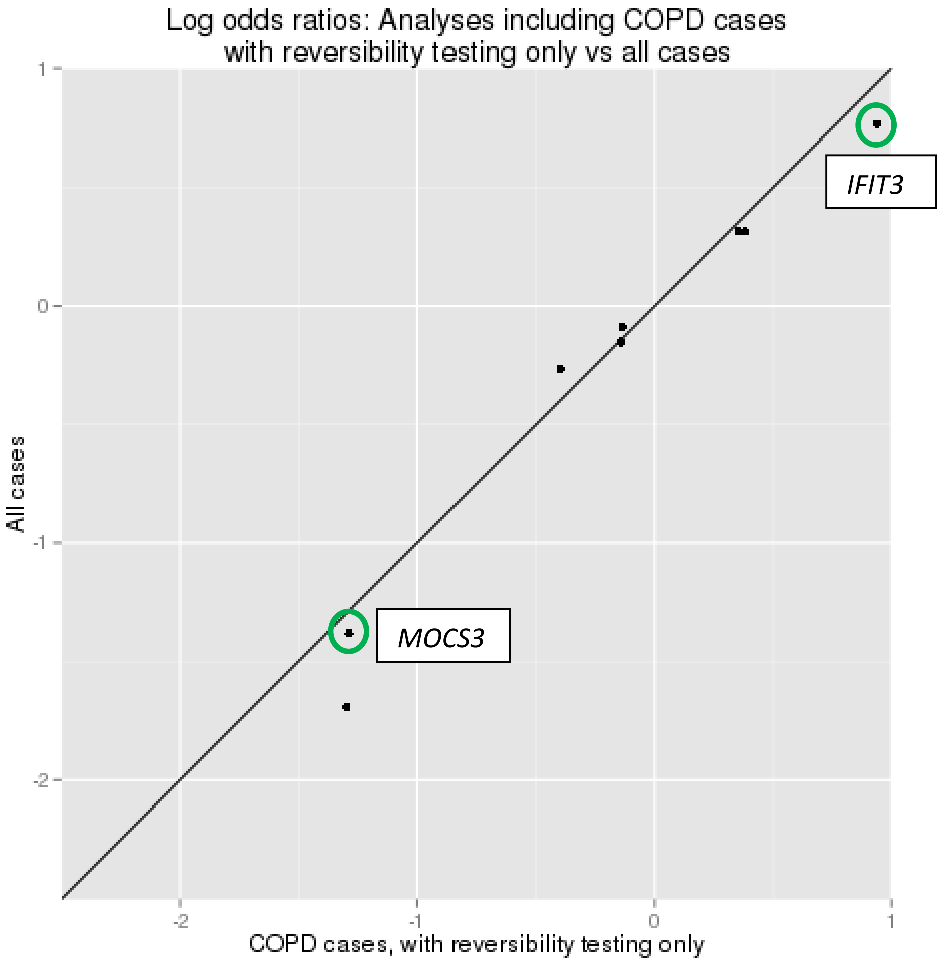

A. Discovery analysis of COPD risk, without adjustment for pack-years

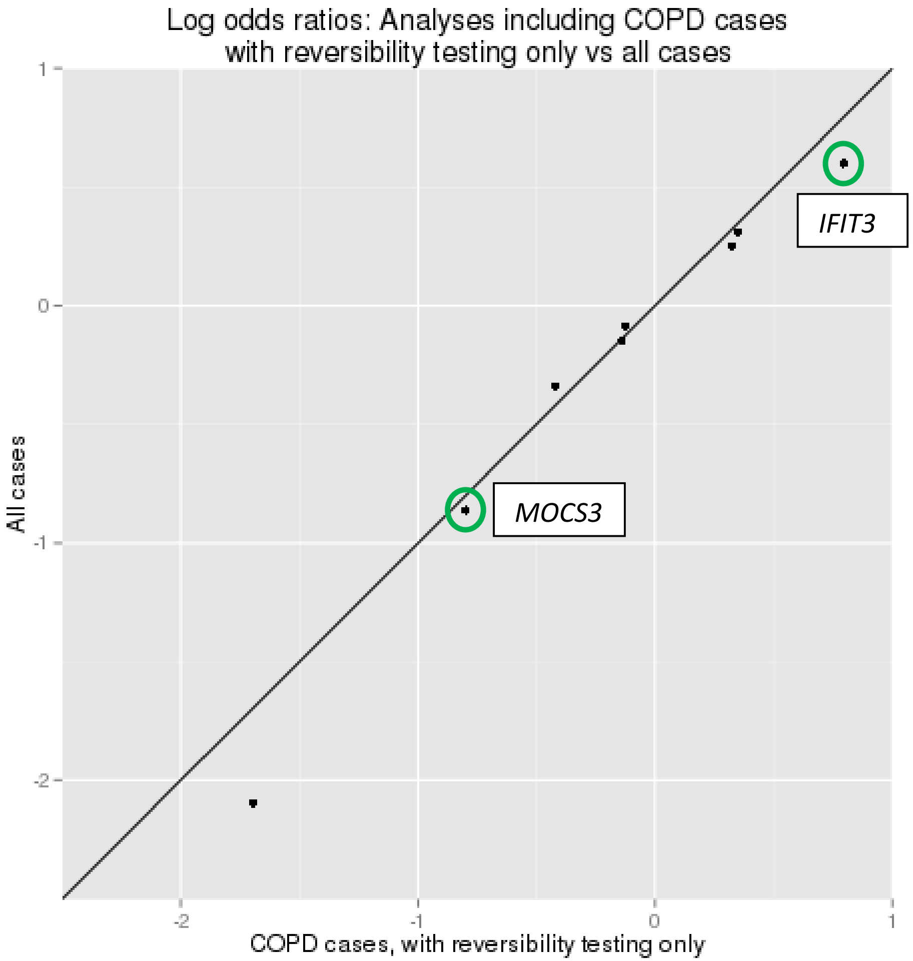

Supplementary Figure 8: Region plots for novel regions associated with COPD risk (A & B) and percent predicted FEV_1 in COPD cases (C).

A. rs7269297 in *MOCS3*¸ associated with COPD risk.

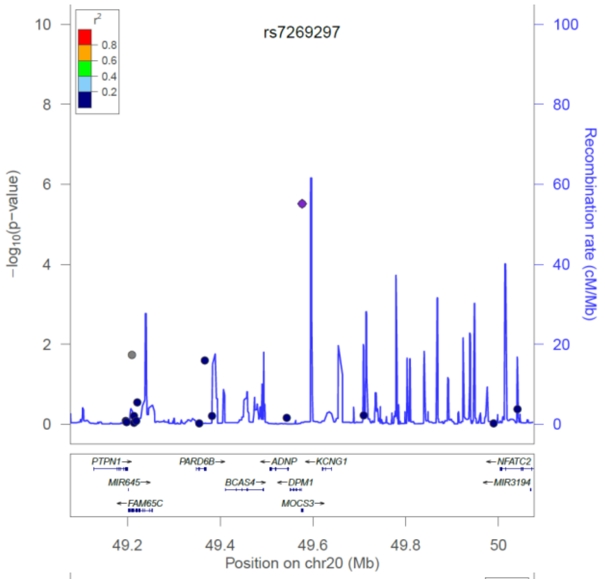

B. rs140549288 in *IFIT3*¸ associated with COPD risk.

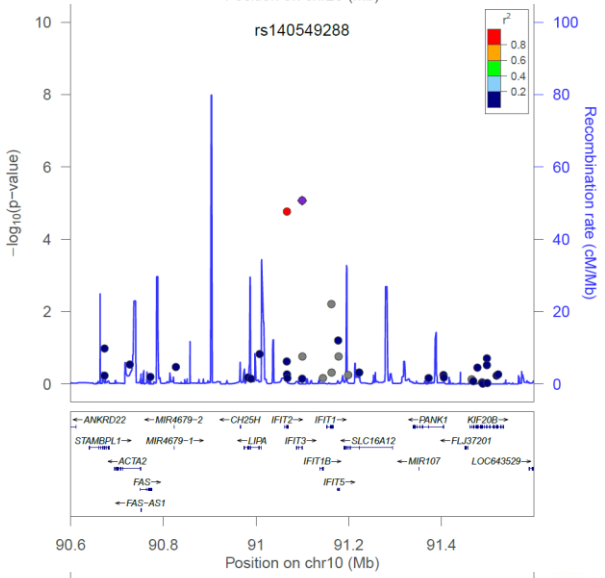

C. rs140198372 in *MOCS3*¸ associated with percent predicated FEV_1 in COPD cases.

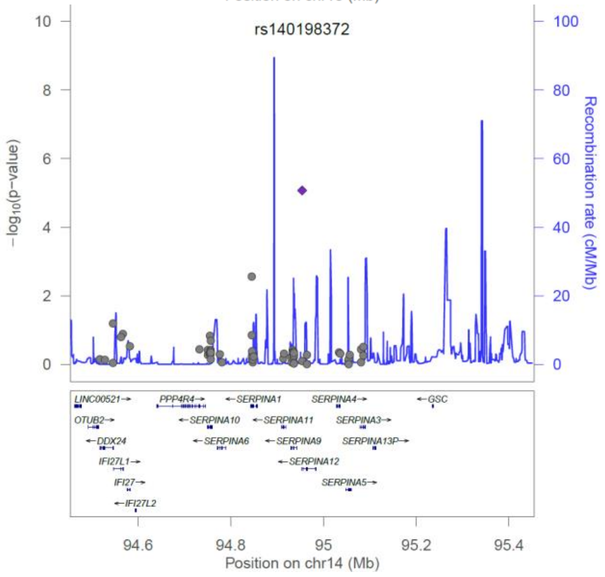

Supplementary methods

Case Collections

Gedling: The Gedling cohort is a general population sample of adults aged 18 to 70 years, recruited in Nottingham in 1991 (n=2,633) in a cross-sectional study of the relationship between diet, asthma, and COPD. Subjects were followed-up in 2000 (n=1346), where they had blood samples taken for DNA extraction (Source Biosciences, UK), completed questionnaires on respiratory symptoms, smoking, and other variables, and had pre-bronchodilator FEV_1 and FVC measurements taken using a calibrated dry bellows spirometer (Vitalograph, Buckingham, UK), recording the best of three satisfactory attempts (1, 2).

EU COPD Gene Scan (EUCOPD): The COPD exome chip study utilised EUCOPD samples recruited from Bristol and Edinburgh. All samples had a clinical diagnosis of stable COPD, with FEV_1/FVC < 70%, percent predicted $FEV_1 \leq 70\%$, according to the Crapo et al. reference equations (3) , no significant reversibility on bronchodilation , and at least 20 pack-years smoking history. Patients were excluded from the study if they had a diagnosis of asthma, established obstructive syndrome, lung cancer, a history of atopy, known alpha-1 antitrypsin deficiency, or a serum alpha-1 antitrypsin level of <1.0g/L, or had experienced an acute exacerbation in the 4 weeks prior to recruitment. (4)

Generation Scotland: Scottish Family Health Study (GS:SFHS):

GS:SFHS is a family-based general population study of approximately 24,000 Scottish individuals aged 18–98 years, recruited through general practices between 2006 and 2011. At baseline, participants had demographic and lifestyle factors collected through questionnaires, blood and saliva samples were collected for DNA extraction and several clinical measures were taken, including spirometry (5). Spirometry was performed three times, without nose clips using the Ndd Easy One Spirometer (Model 2001). The maximum values of FVC and FEV_1 were used in the analyses.

Participants were excluded from spirometry testing if <12 or >24 weeks pregnant; had stroke or heart attack in previous year; used inhaler and did not have it with them; had collapsed lung, flu, severe cold, chest infection or any surgery in previous month; had detached retina in previous 3 months.

British Regional Heart Study (BRHS): The BRHS includes 7735 men, aged 40-59 who were recruited from 24 towns across the UK between 1978 and 1980, with a follow-up assessment in 1998-2000 (n=4252). Participants periodically completed questionnaires which including questions on lifestyle, medication use and respiratory symptoms. At baseline and follow-up examinations, participants had

lung function measurements taken (6). Measures used in this study were from the follow-up assessment, where a minimum of 3 spirometry measurements were obtained standing and without noseclips, using a Vitalograph Compact II instrument (Vitalograph Ltd, Buckingham,UK). FEV_1 and FVC were recorded for the best test, which was defined in accordance with American Thoracic Society recommendations(7). Blood samples for DNA extraction were taken at the follow-up examination (6).

British Women's Health and Heart Study (BWHHS): The BWHHS recruited 4286 women aged 60-79 between 1999 and 2001. Participants were recruited from 23 centres in the UK (22 centres common with BRHS study) and were matched in terms of town and age to BRHS participants. At baseline Clinical measurements were made on 3995 with 3923 providing some blood. DNA was also extracted for genotyping from the blood samples. Baseline data collection took place between April 1999 and March 2001. Participants also completed questionnaires on lifestyle and medical history. (8) Participants performed spirometry measurements, using digital meter Vitalograph, until three reproducible blows were achieved(within 5% of maximum FVC produced). The blow with the highest sum of FVC and FEV_1 was used in the analyses.

UK COPD Cohort (UKCOPD): COPD subjects were recruited from five UK centres based on physician- and spirometry-defined COPD (FEV1/FVC <70%; FEV1 <80%), Caucasian, >40 yr old, smokers with >10 pack-yr history (9). Ethical approval was obtained from the Multicenter Research Ethics Committee (MREC/99/4/001) and informed consent from all subjects was obtained.

Hertfordshire Cohort Study (HCS):HCS recruited men and women from Hertfordshire, born between 1931 and 1939. At baseline, study participants completed questionnaires, including demographics, medical history, and details of respiratory symptoms. 2997 recruited individuals attended a clinic, at which they had lung function measurements and blood taken for DNA extraction (10). Spirometry measurements were taken three times with participants seated and without noseclips, using a Micro Spirometer (Micro Medical Ltd). The highest FEV_1 and FVC values from satisfactory manoeuvres (not necessarily from the same blow) were used in the analyses.

COPD-BEAT (Biomarkers to target Antibiotic & Systemic Corticosteroid therapy in COPD exacerbations): Recruited individuals with COPD in two 1 year studies in series with part 1 a one year observational study and part 2 where subjects were randomised to either standard care or targeted care following part 1. Subjects in the targeted therapy arm were further randomised to receive either prednisolone or matching placebo determined by the biomarker- the blood eosinophil count. Subjects were assessed at 3 monthly stable visits, at exacerbation onset and 2 and 6 week

recovery. Assessments included symptoms, health status, airway inflammation, lung function, microbiology and virology.

Nottingham COPD Study (NottCOPD): Recruited individuals between 40-85 years of age who had smoked at least 10 pack years and were Caucasian. A diagnosis of COPD was made on basis of clinical assessment and spirometry when the subject was at clinical stability (> 4 weeks from any exacerbation or change in medication). Spirometry was performed (pre and post bronchodilator) on a Micromedical MK6 (Micro Medical Ltd, UK) with the best of three satisfactory and repeatable attempts used in the analysis (11). Blood samples for DNA extraction were taken at the same visit.

Nottingham Smokers: The Nottingham Smokers cohort recruited individuals in Nottingham who were Caucasian, over 40 years of age and with at least 10 pack years smoking history. Participants had spirometry measured at enrolment using a MicroLab ML3500 spirometer (Micro Medical Ltd, UK) with the best of three satisfactory attempts used in the analyses.

English Longitudinal Study of Aging (ELSA):ELSA is a longitudinal study of individuals aged over 50 and their partners, who had previously responded to the Health Survey of England between 1998 and 2001. Individuals were recruited in 2002-3, with participants having follow-up interviews every two years. Spirometric measures used in these analyses were taken at the third wave of interviews (2006-2007).

GoTARDIS Study: The GoTARDIS study recruited individuals with COPD in Dundee, Scotland who were being managed by the community respiratory care network, through the TARDIS database. All COPD patients in Dundee attend annual meetings at their GP practice with a respiratory nurse, where spirometry, disease activity and treatment information are recorded and stored in the central TARDIS database. Patients were recruited through their General Practitioners and provided consent for linkage of their genetic to their medical records. Participants provided saliva samples for DNA extraction using Oragene saliva collection kits.

<u>Genotype calling and Quality Control (QC) of exome array genotype data</u>

Genotypes were called using Illumina's Gencall algorithm in Genomestudio (12), then SNPs and samples with >90% missing data were removed. Subsequently, samples with a call rate < 98%, heterozygosity rate>3 standard deviations (SD) from mean (calculated separately using SNPs with MAF>=1% and SNPs with MAF<1%), gender mismatches, and duplicates were excluded. Additionally, samples with an excess of singleton SNPs (those who were the only individual to have the minor allele for >50 SNPs), and samples whose mean probe intensity across all autosomal SNPs was outlying were also excluded. Ancestry principal components analysis (PCA) was carried out using

EIGENSTRAT(13), with a subset of Linkage disequilibrium (LD) pruned HapMap 3 CEU SNPs with MAF>1% and call rate>99%; any individuals >4SD from the sample mean for either of the first two principal components were excluded. Additionally, SNPs were excluded if they showed differential rates of missingness in cases and GS:SFHS controls ($P<10^{-4}$).

Following these exclusions, missing genotypes were recalled using zCall (14) and a second stage of QC was carried out. SNPs with call rate<99% or which deviated from Hardy Weinberg Equilibrium ($P<10^{-4}$) were excluded, along with samples with call rate < 99%, and heterozygosity outliers (>3SD from mean). To eliminate variants that were subject to genotyping batch effects, we tested for associations between genotype and sample collection, separately in cases and controls; any SNP showing association ($P<10^{-5}$) was excluded.

Discovery exome gene-based analysis

Variants were annotated to genes using ANNOVAR (15) on the basis of the GRCh37/hg19 database and all exonic variants were included in the analyses. Analyses of COPD risk and severity of airflow limitation were undertaken using SKAT-O (16), with covariate adjustments analogous to the single variant analyses and using default beta distribution weightings (weight for j^{th} variant: $\beta(MAF_j;\ 1,\ 25)$). We filtered gene-based results to only include genes with at least two SNPs with a MAF<5%, and with a cumulative MAF>0.05%. To further evaluate notable gene based signals, we utilised a "drop-one" analysis. This involved recalculating the SKAT-O P-value when individual SNPs are sequentially excluded from the test. If the SKAT-O P-value was considerably attenuated by the removal of a particular SNP, this would indicate that the SKAT-O signal was likely to be largely influenced by that individual SNP, rather than variants within that gene as a whole. Applying a Bonferroni correction for the number of genes tested results in a significance level of $P<3.5\times10^{-6}$; we took forward genes with $P<10^{-5}$ for replication analyses in UK BiLEVE.

Custom content design, genotyping and QC of genotype data

All 3487 cases and 1032 GS:SFHS controls were genotyped using the Illumina Human Exome BeadChip, with additional custom content, selected as follows:53,300 SNPs were selected as showing association with FEV_1 and/or FEV_1/FVC with P<0.01 from the discovery stage of a meta-analysis of 48,000 individuals (17). Each SNP was assigned the P value for the trait with which it showed the strongest association and SNPs were then ranked by significance. The SNPs were then LD pruned such that for SNPs with $P<10^{-4}$, all SNP within 500kb and with $r^2>0.5$ with the most

significant SNP were removed and for SNPs with $10^{-4}<P<0.01$, SNPs within 500kb and with r2>0.2 were removed. This method of LD pruning was intended to result in higher coverage of SNPs in regions showing slightly stronger evidence of association. Sample exclusions were carried out identically to the exome analysis. SNP exclusions were undertaken to remove those with a call rate < 95%, deviated from HWE ($P<10^{-4}$) or were monomorphic.

Additional controls for the custom content analyses came from 1958BC and Busselton Health Study (BHS) genotyped using the Affymetrix 500k, or Illumina 550k, 660k, 1M 610-Quad or 660w-Quad SNP genotyping platforms and then imputed to the 1000 Genomes Project Phase 1 (1000G) reference panel (18). Both the 1958BC and BHS controls genotype data were phased using MACH v.1.0.18 (19), with 1958BC imputed using Minimac v.2012.11.16 and BHS imputed using Minimac v.2012.10.3 (20). Post-imputation, SNPs were excluded if they had low imputation quality ($R^2<0.3$) in either the 1958BC or BHS data. We additionally undertook control set comparisons for all custom content SNPs to identify possible batch effects (comparison of GS:SFHS controls vs all other controls). We subsequently removed any SNP showing association to control set with P<0.01. The 1958BC and BHS control samples used in these analyses are summarised in Supplementary Table 4.

Supplementary Table 4: Characteristics of the control samples used in the Custom Content analyses (Characteristics of cases and GS:SFHS controls shown in Table 1 of manuscript).

Sample Collection	n	Sex	Age	Percent Predicted FEV_1	FEV_1/FVC	Pack-years	
		Male, n (%)	Mean (SD)	Mean (SD)	Mean (SD)	Samples with data (n)	Mean (SD)
**Controls (Custom Content analysis: total n=3262, with pack-years n=2252)**							
British 1958 Birth Cohort (1958BC)	1585	875 (55.2%)	44 (0)	100.13 (13.40)	0.810 (0.060)	1160	14.4 (13.35)
Busselton Health Study (BHS)	716	403 (56.3%)	56.53 (11.26)	99.43 (11.38)	0.78 (0.044)	131	34.39 (30.91)

Custom content single variant analyses

Single variant associations with the custom content SNPs and both COPD risk and severity of airflow limitation were undertaken equivalently to the exome analyses. The custom content analysis included only SNPs with *a priori* evidence of association with lung function, thus we used a threshold of $P<10^{-3}$ for selecting SNPs for replication analyses in UK BiLEVE. We set a Bonferroni corrected significance level for replication, for the number of SNPs in novel regions taken forward to replication (P<0.0125 for analysis of COPD risk; P<0.010 for analysis of airflow limitation severity).

Replication and meta-analysis with UK BiLEVE data

The 4231 cases with airflow limitation indicative of COPD and 8979 controls from UK BiLEVE contributing to the meta-analysis, were selected based on their % predicted FEV_1 values, calculated using reference equations derived using healthy never smokers in the whole of UK Biobank. For association testing, percent predicted FEV_1 was recalculated using the NHANES III spirometric reference equations (21) for consistency with the exome discovery analyses. All selected COPD cases met GOLD 2 criteria (FEV_1/FVC<0.7 and % predicted FEV_1<80%) under both reference equations.

SNP associations with risk of COPD were carried out using a logistic regression model, implemented in Plink v1.07 (22) adjusting for age, sex and pack-years and assuming an additive genetic model. In the analysis of severity of airflow limitation, associations with untransformed percent predicted FEV_1, in cases were tested using a linear regression model, with adjustment for pack-years.

The genomic inflation factor (λ) was calculated for the exome array and UK BiLEVE analyses and where λ>1, genomic control was applied, adjusting the standard errors of effect estimates accordingly. All SNPs were oriented to the same strand, with consistent coded alleles. Effect estimates and standard errors were combined across the two analyses using an inverse-variance–weighting meta-analysis. λ was calculated for the pooled effect estimates and genomic control was applied again where λ>1. Meta-analysis statistics and figures were produced using R version 3.1.1.

For the replication of associations identified in the discovery exome analyses, a look-up of the UK BiLEVE and meta-analysis results was undertaken. Replication of associations identified through the custom content analyses was undertaken in the same way, where SNPs were genotyped in the UK BiLEVE samples. Where a SNP was not directly genotyped, additional analyses was carried out using imputed data: UK BiLEVE samples were imputed to a combined 1000G (18) and UK10K Project (23) reference panel. Following imputation, SNPs were excluded if they had imputation INFO score ≤0.5

or minor allele count (MAC)<3. Associations were carried out for relevant SNPs using SNPTEST v2.5b4 (24).

Supplementary Results

Discovery exome gene-based analysis

In the gene-based analyses of COPD risk, *PRICKLE1* was the only gene to reach the $P<10^{-5}$ significance level ($P=1.968\times10^{-6}$, unadjusted analysis). The SKAT-O test included three SNPs within this gene, however "drop-one" analyses showed the signal to be entirely driven by rs3827522 (MAF=0.4%), the SNP identified in the single variant analysis (Supplementary Table 5). The analyses of severity of airflow limitation identified no associated genes with $P<10^{-5}$.

Supplementary Table 5: Risk of COPD single variant association results of SNPs included in SKAT-O test of *PRICKLE1* (SKAT-O test P-value= 1.968×10^{-6})

		Risk of COPD with pack-years adjustment				Unadjusted analysis of risk of COPD				Drop-one result	
		MAF (MAC)		Association result		MAF (MAC)		Association result			
rs no.	Coded Allele	Cases (n=2517)	Controls (n=3889)	OR	P	Cases (n=3226)	Controls (n=4784)	OR	P	SKAT-O Analysis utilising all SNPs in *PRICKLE1*	P-value of SKAT-O Analysis if SNP removed
rs3827522	A	0.22% (11)	0.35% (27)	0.628	1.38×10^{-3}	0.22% (14)	0.0048 (46)	0.123	1.03×10^{-7}	Unadjusted<br>P=1.968×10^{-6}<br><br>Pack-years adjusted<br>P=2.086×10^{-3}	unadjusted:P=0.0484;<br>adjusted: P=0.0258
rs146199468	G	0.02% (1)	0.00% (0)	-	-	0.02% (1)	0.00 (0)	-	-		unadjusted:P=1.973×10^{-6};<br>adjusted: P=4.347×10^{-3}
rs79087668	T	0.20% (10)	0.32% (25)	0.617	0.999	0.20% (13)	0.0030 (29)	0.364	4.44×10^{-2}		unadjusted:P=1.693×10^{-9};<br>adjusted: P=1.367×10^{-3}

Custom content single variant association analysis

After exclusions, 3226 case samples, 3262 controls and 2489 SNPs were included in the custom content analyses of COPD risk and severity of airflow limitation. The strongest signal identified in these analyses was for an association with COPD risk at the previously reported 15q25 region (rs2036527 near *CHRNA5,* Supplementary Figure 9 and Supplementary Table 6). There were two additional SNPs within the 15q25 region showing association with COPD risk; conditional analyses confirmed that these did not represent an independent signal to the sentinel SNP (Supplementary Table 7). Although an additional four SNPs showed association ($P<10^{-3}$) with COPD risk and 5 SNPs showed association ($P<10^{-3}$) with severity of airflow obstruction in cases (Supplementary Figure 10 and Supplementary Table 8), none were further replicated in the UK BiLEVE study.

Supplementary Figure 9: A) Custom content analysis of COPD risk, with pack-years adjustment (SNPs with $P<10^{-3}$ highlighted). B) Custom content analysis of COPD risk, without pack-years adjustment (SNPs with $P<10^{-3}$ highlighted).

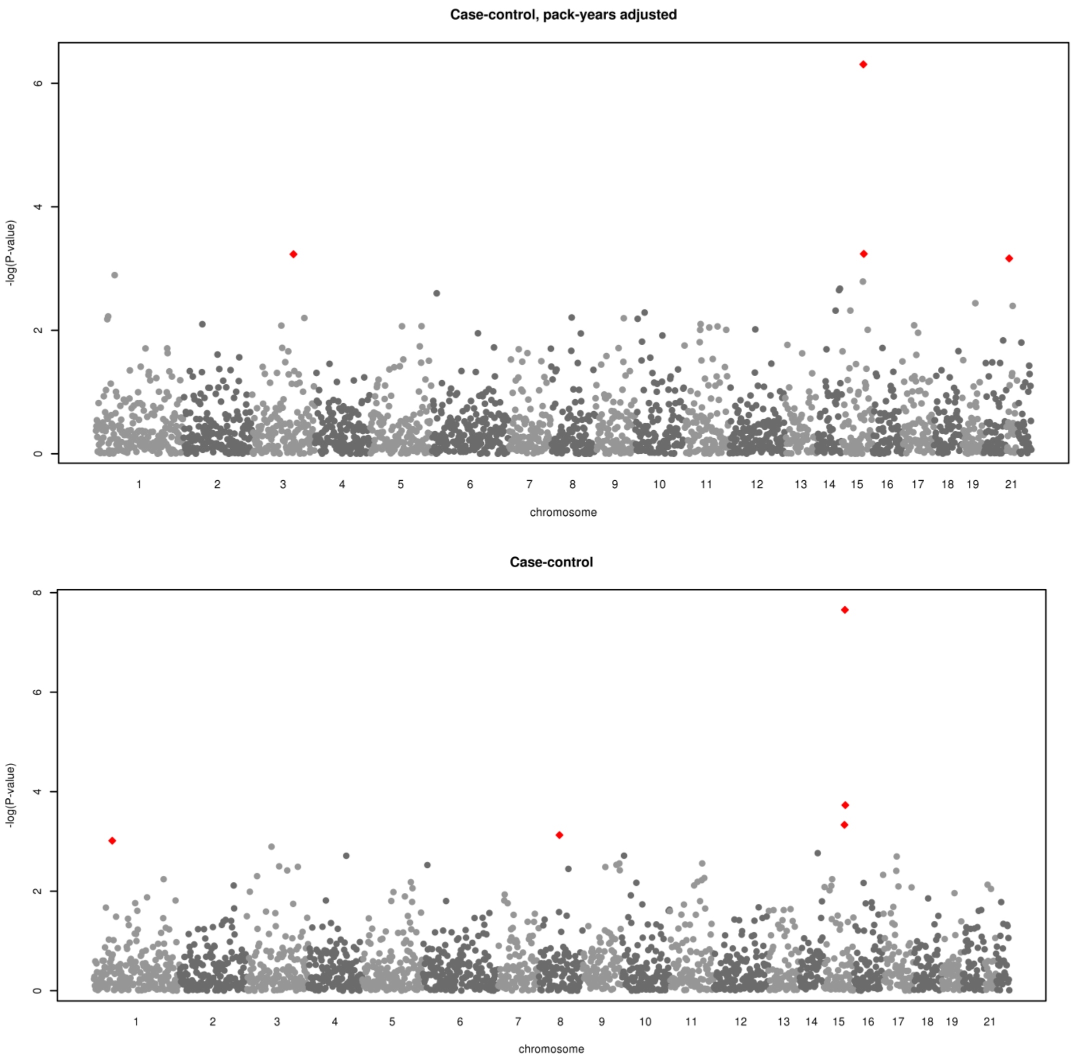

Supplementary Figure 10: A) Custom content analysis of severity of airway limitation, with pack-years adjustment (SNPs with $P<10^{-3}$ highlighted). B) Custom content analysis of severity of airway limitation, without pack-years adjustment (SNPs with $P<10^{-3}$ highlighted).

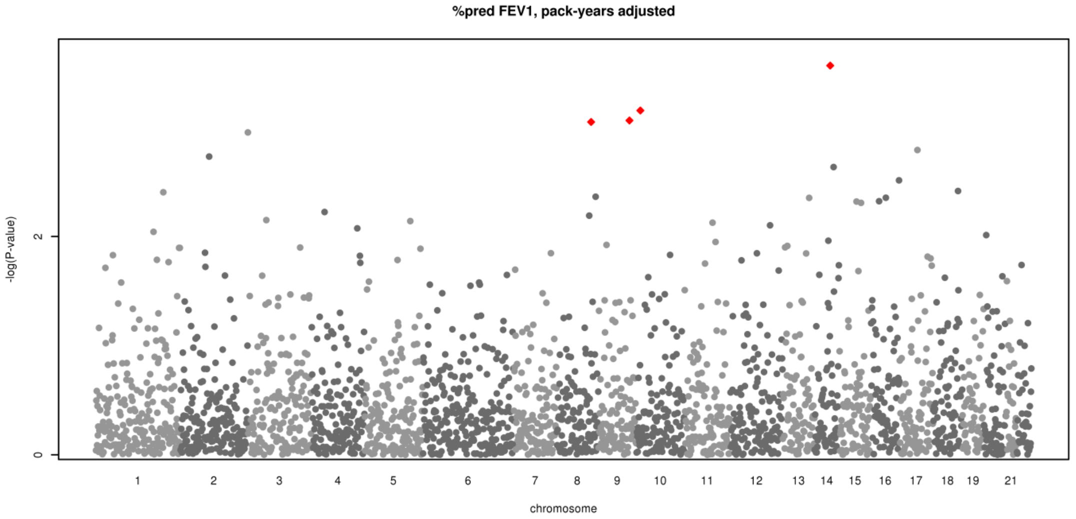

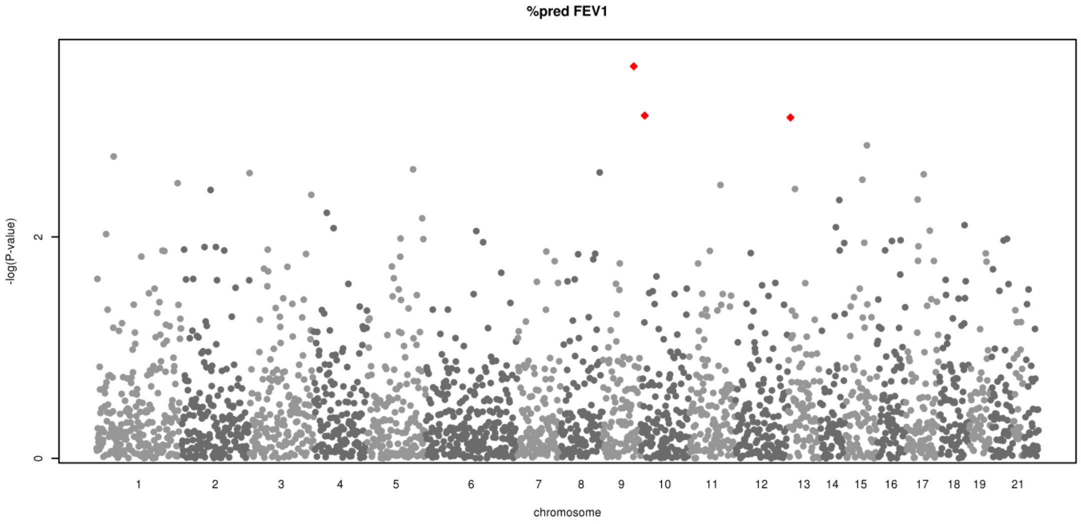

Supplementary Table 6: Top associations ($P<10^{-3}$) from custom content analyses of COPD risk, with and without adjustment for pack-years smoking and replication in UK BiLEVE.

					Discovery pack-years adjusted analysis (2517 cases, 2252 controls)			Discovery unadjusted analysis (3226 cases, 3262 controls)			UK BiLEVE pack-years adjusted analysis (4231 cases, 8979 controls)			
rs no.	CHR	Position	Coded Allele	Gene	MAF (MAC)	OR	P*	MAF (MAC)	OR	P*	Imputation quality (R2) †	MAF (MAC)	OR	P*
rs7549174	1	28319484	G	*EYA3* (intronic)	30.8% (2934)	0.8066	1.27×10^{-3}	31.0% (4023)	0.8375	**9.68×10^{-4}**	0.993	31.0% (8183)	1.0133	0.665
rs4622901	3	132697086	A	*near TMEM108* (intergenic)	34.5% (3290)	0.8021	**5.86×10^{-4}**	35.4% (4588)	0.8618	3.83×10^{-3}	0.999	36.0% (9504)	0.9903	0.740
rs10957070	8	59712593	G	near *TOX* (intergenic)	26.3% (2504)	1.212	6.21×10^{-3}	25.9% (3364)	1.212	**7.45×10^{-4}**	0.989	25.9% (6833)	0.9884	0.717
rs2036527	15	78851615	A	near *CHRNA5* (intergenic)	31.5% (3006)	1.38	**4.94×10^{-7}**	35.3% (4581)	1.338	**2.22×10^{-8}**	0.995	34.8% (7631)	1.029	0.077
rs2510527	21	31577206	A	*near CLDN8* (intergenic)	5.9% (560)	0.6431	**6.86×10^{-4}**	5.8% (752)	0.7541	7.41×10^{-3}	0.997	5.7% (1495)	0.9901	0.870

***P-values in bold significant at $P<10^{-3}$ level**
†SNPs which were directly genotyped have imputation quality of NA.

Supplementary Table 7: Risk of COPD associations within *CHRNA5* region (Custom content analysis signals rs8042238 and rs569207), conditional on rs2036527.

	Risk of COPD with pack-years adjustment				Unadjusted analysis of risk of COPD			
	Single SNP association		Association conditional on rs2036527		Single SNP association		Association conditional on rs2036527	
rs no.	OR	P	OR	P	OR	P	OR	P
rs8042238	0.8168	1.63×10^{-3}	0.934	0.345	0.8351	4.64×10^{-4}	0.939	0.277
rs569207	0.768	5.79×10^{-3}	0.867	0.083	0.7937	1.86×10^{-4}	0.885	0.065

Supplementary Table 8:Top associations ($P<10^{-3}$) in custom content analysis of severity of airflow limitation, with and without adjustment for pack-years smoking and replication in UK BiLEVE.

					Severity of airflow limitation, adjusted for pack-years (n=2517)			Unadjusted analysis of severity of airflow limitation (n=3226)			UK BiLEVE pack-years adjusted analysis (4231 cases, 8979 controls)			
rs no.	CHR	Position	Coded Allele	Gene	MAF (MAC)	Beta	P*	MAF (MAC)	Beta	P*	Imputation quality (R2) †	MAF (MAC)	Beta	P*
rs7816350	8	126289605	G	*NSMCE2* (intronic)	12.1% (611)	2.40	**8.93×10^{-4}**	12.0% (773)	1.59	$1.42\text{x}10^{-2}$	NA	11.5% (974)	0.006	0.9879
rs12235542	9	125234354	A	*OR1J2* (intronic)	44.8% (2254)	1.57	**8.65×10^{-4}**	44.8% (2887)	1.53	**2.87×10^{-4}**	0.979	45.0% (3811)	-0.021	0.3349
rs1923394	10	4307011	A	near *LINC00702* (intergenic)	15.7% (792)	2.23	**7.01×10^{-4}**	16.0% (1033)	1.95	**8.01×10^{-4}**	0.982	15.6% (1320)	-0.0547	0.0667
rs1220574	13	24757444	A	*SPATA13* (intronic)	0.02% (1)	-41.93	1.26×10^{-2}	0.05% (3)	-32.67	**8.34×10^{-4}**	0.557	0.16% (14)	0.411	0.2538
rs1705662	14	84282660	A	intergenic	15.8% (795)	2.38	**2.72×10^{-4}**	15.6% (1006)	1.55	$8.23\text{x}10^{-3}$	0.990	15.6% (1270)	0.0233	0.4365

***P-values in bold significant at $P<10^{-3}$ level**
†SNPs which were directly genotyped have imputation quality of NA.

References

1. McKeever TM, Scrivener S, Broadfield E, Jones Z, Britton J, Lewis SA. Prospective Study of Diet and Decline in Lung Function in a General Population. Am J Respir Crit Care Med. 2002 05/01; 2015/07;165(9):1299-303.

2. Wain LV, Odenthal-Hesse L, Abujaber R, Sayers I, Beardsmore C, Gaillard EA, et al. Copy Number Variation of the Beta-Defensin Genes in Europeans: No Supporting Evidence for Association with Lung Function, Chronic Obstructive Pulmonary Disease or Asthma. PLoS ONE. 2014 01/03;9(1):e84192.

3. Crapo R, Morris A, Gardner R. **Reference spirometric values using techniques and equipment that meet ATS recommendations.**. Am Rev Respir Dis. 1981;123 (6):659-64.

4. Chappell S, Daly L, Morgan K, Guetta Baranes T, Roca J, Rabinovich R, et al. Cryptic haplotypes of SERPINA1 confer susceptibility to chronic obstructive pulmonary disease. Hum Mutat. 2006;27(1):103-9.

5. Smith BH, Campbell A, Linksted P, Fitzpatrick B, Jackson C, Kerr SM, et al. Cohort Profile: Generation Scotland: Scottish Family Health Study (GS:SFHS). The study, its participants and their potential for genetic research on health and illness. International Journal of Epidemiology. 2013 June 01;42(3):689-700.

6. Walker M, Whincup P, Shaper A. The British Regional Heart Study 1975–2004. International Journal of Epidemiology. 2004 December 01;33(6):1185-92.

7. Wannamethee SG, Shaper AG, Whincup PH. Body fat distribution, body composition, and respiratory function in elderly men. The American Journal of Clinical Nutrition. 2005 November 01;82(5):996-1003.

8. Lawlor DA, Bedford C, Taylor M, Ebrahim S. Geographical variation in cardiovascular disease, risk factors, and their control in older women: British Women's Heart and Health Study. Journal of Epidemiology and Community Health. 2003 February 01;57(2):134-40.

9. Stewart CE, Hall IP, Parker SG, Moffat MF, Wardlaw AJ, Connolly MJ, et al. PLAUR polymorphisms and lung function in UK smokers. BMC Med Genet. 2009 Oct 31;10:112,2350-10-112.

10. Syddall H, Aihie Sayer A, Dennison E, Martin H, Barker D, Cooper C, et al. Cohort Profile: The Hertfordshire Cohort Study. International Journal of Epidemiology. 2005 December 01;34(6):1234-42.

11. Miller MR, Hankinson J, Brusasco V, Burgos F, Casaburi R, Coates A, et al. Standardisation of spirometry. European Respiratory Journal. 2005 August 01;26(2):319-38.

12. Illumina Inc. Illumina GenCall Data Analysis Software. 2005.

13. Price AL, Patterson NJ, Plenge RM, Weinblatt ME, Shadick NA, Reich D. Principal components analysis corrects for stratification in genome-wide association studies. Nat Genet. 2006 print;38(8):904-9.

14. Goldstein JI, Crenshaw A, Carey J, Grant GB, Maguire J, Fromer M, et al. zCall: a rare variant caller for array-based genotyping: Genetics and population analysis. Bioinformatics. 2012 October 01;28(19):2543-5.

15. Wang K, Li M, Hakonarson H. ANNOVAR: functional annotation of genetic variants from high-throughput sequencing data. Nucleic Acids Research. 2010 September 01;38(16):e164-.

16. Lee S, Emond MJ, Bamshad MJ, Barnes KC, Rieder MJ, Nickerson DA, et al. Optimal Unified Approach for Rare-Variant Association Testing with Application to Small-Sample Case-Control Whole-Exome Sequencing Studies. The American Journal of Human Genetics. 2012 8/10;91(2):224-37.

17. Soler Artigas M, Loth DW, Wain LV, Gharib SA, Obeidat M, Tang W, et al. Genome-wide association and large-scale follow up identifies 16 new loci influencing lung function. Nat Genet. 2011 print;43(11):1082-90.

18. The 1000 Genomes Project Consortium. An integrated map of genetic variation from 1,092 human genomes. Nature. 2012 11/01;491(7422):56-65.

19. Li Y, Willer CJ, Ding J, Scheet P, Abecasis GR. MaCH: using sequence and genotype data to estimate haplotypes and unobserved genotypes. Genet Epidemiol. 2010;34(8):816-34.

20. Howie BN, Donnelly P, Marchini J. A Flexible and Accurate Genotype Imputation Method for the Next Generation of Genome-Wide Association Studies. PLoS Genet. 2009 06/19;5(6):e1000529.

21. HANKINSON J, ODENCRANTZ J, FEDAN K. Spirometric Reference Values from a Sample of the General U.S. Population. Am J Respir Crit Care Med. 1999 01/01; 2014/03;159(1):179-87.

22. Purcell S, Neale B, Todd-Brown K, Thomas L, Ferreira MAR, Bender D, et al. PLINK: A Tool Set for Whole-Genome Association and Population-Based Linkage Analyses. The American Journal of Human Genetics. 2007 9;81(3):559-75.

23. Huang J, Howie B, McCarthy S, Memari Y, Walter K, Min J, et al. Improved imputation of low-frequency and rare variants using the UK10K haplotype reference panel. Nature Communications. 2015;6(2015/09/14/online).

24. Marchini J, Howie B, Myers S, McVean G, Donnelly P. A new multipoint method for genome-wide association studies by imputation of genotypes. Nat Genet. 2007;39(7):906-13.
